# Supplementary material for: Motivational interviewing to support modifiable risk factor change in individuals at increased risk of cardiovascular disease: A systematic review and meta-analysis
Source: PLoS One. 2020 Nov 11;15(11):e0241193. doi: 10.1371/journal.pone.0241193 (PMC7657493; doi:10.1371/journal.pone.0241193)
Supplement: S1 Appendix — (DOCX) [file pone.0241193.s001.docx]

# S1 Appendix supplementary information.

**Table S1. PRISMA checklist**

| Section/topic | Item No | PRISMA Checklist item | Reported on page No |
| --- | --- | --- | --- |
| **Title** | | | |
| Title | 1 | Identify the report as a systematic review, meta-analysis, or both | 1 |
| **Abstract** | | | |
| Structured summary | 2 | Provide a structured summary including, as applicable, background, objectives, data sources, study eligibility criteria, participants, interventions, study appraisal and synthesis methods, results, limitations, conclusions and implications of key findings, systematic review registration number | 1-4 |
| **Introduction** | | | |
| Rationale | 3 | Describe the rationale for the review in the context of what is already known | 5 |
| Objectives | 4 | Provide an explicit statement of questions being addressed with reference to participants, interventions, comparisons, outcomes, and study design (PICOs) | 6-7 |
| **Methods** | | | |
| Protocol and registration | 5 | Indicate if a review protocol exists, if and where it can be accessed (such as web address), and, if available, provide registration information including registration number | 7 |
| Eligibility criteria | 6 | Specify study characteristics (such as PICOs, length of follow-up) and report characteristics (such as years considered, language, publication status) used as criteria for eligibility, giving rationale | 8-9 |
| Information sources | 7 | Describe all information sources (such as databases with dates of coverage, contact with study authors to identify additional studies) in the search and date last searched | 8 |
| Search | 8 | Present full electronic search strategy for at least one database, including any limits used, such that it could be repeated | S1 Appendix |
| Study selection | 9 | State the process for selecting studies (that is, screening, eligibility, included in systematic review, and, if applicable, included in the meta-analysis) | 8-9 |
| Data collection process | 10 | Describe method of data extraction from reports (such as piloted forms, independently, in duplicate) and any processes for obtaining and confirming data from investigators | 10 |
| Data items | 11 | List and define all variables for which data were sought (such as PICOs, funding sources) and any assumptions and simplifications made | 10-11 (see S1 Appendix table S4) |
| Risk of bias in individual studies | 12 | Describe methods used for assessing risk of bias of individual studies (including specification of whether this was done at the study or outcome level), and how this information is to be used in any data synthesis | 11 |
| Summary measures | 13 | State the principal summary measures (such as risk ratio, difference in means). | 11-12 |
| Synthesis of results | 14 | Describe the methods of handling data and combining results of studies, if done, including measures of consistency (such as I2) for each meta-analysis | 12 |
| Risk of bias across studies | 15 | Specify any assessment of risk of bias that may affect the cumulative evidence (such as publication bias, selective reporting within studies) | 11 |
| Additional analyses | 16 | Describe methods of additional analyses (such as sensitivity or subgroup analyses, meta-regression), if done, indicating which were pre-specified | 12 |
| **Results** | | | |
| Study selection | 17 | Give numbers of studies screened, assessed for eligibility, and included in the review, with reasons for exclusions at each stage, ideally with a flow diagram | 12 |
| Study characteristics | 18 | For each study, present characteristics for which data were extracted (such as study size, PICOs, follow-up period) and provide the citations | 12 |
| Risk of bias within studies | 19 | Present data on risk of bias of each study and, if available, any outcome-level assessment (see item 12). | 19, 32 |
| Results of individual studies | 20 | For all outcomes considered (benefits or harms), present for each study (a) simple summary data for each intervention group and (b) effect estimates and confidence intervals, ideally with a forest plot | 32 |
| Synthesis of results | 21 | Present results of each meta-analysis done, including confidence intervals and measures of consistency | 32 |
| Risk of bias across studies | 22 | Present results of any assessment of risk of bias across studies (see item 15) | 12 |
| Additional analysis | 23 | Give results of additional analyses, if done (such as sensitivity or subgroup analyses, meta-regression [see item 16]) | 23,25 |
| **Discussion** | | | |
| Summary of evidence | 24 | Summarise the main findings including the strength of evidence for each main outcome; consider their relevance to key groups (such as health care providers, users, and policy makers) | 35-37 |
| Limitations | 25 | Discuss limitations at study and outcome level (such as risk of bias), and at review level (such as incomplete retrieval of identified research, reporting bias) | 35-39 |
| Conclusions | 26 | Provide a general interpretation of the results in the context of other evidence, and implications for future research | 39-40 |
| **Funding** | | | |
| Funding | 27 | Describe sources of funding for the systematic review and other support (such as supply of data) and role of funders for the systematic review | na |

**Table S2. Review protocol.**

| Review title | Effectiveness of primary preventive programmes using motivational interviewing to support lifestyle modification; a systematic literature review and meta-analysis. |
| --- | --- |
| Funded proposal | NA |
| Review question | Is motivational interviewing effective in supporting adults at increased risk of CVD to make healthy lifestyle changes to reduce cardiovascular risk?  What are the characteristics of effective interventions using motivational interviewing? |
| Searches including limiters. | CINAHL Complete (EBSCO)  APA PsycINFO (EBSCO)  Academic Search Ultimate (EBSCO)  Cochrane Central Register of Controlled Trials (EBSCO)  E-Journals (EBSCO)  MEDLINE Complete (EBSCO)  Psychology Database  PubMed  PLOS  DynaMed Plus  Date: February 2013- March 2020  Type: Peer reviewed journals only  Language: Published in English language  Age: Adults |
| Population. | Adult participants over the age of eighteen, of both genders, representing all ethnicities and employment status, having at least one or more modifiable risk factor/s. |
| Intervention. | Preventive programmes, which made use of motivational interviewing principles with the aim to support changes in modifiable risk factors amongst participants with one or more modifiable risk/s. |
| Comparator. | Standard health information/usual general practice/no intervention. |
| Types of study design to be included. | Randomised controlled trials |
| Main outcome. | The primary outcome in this review is to determine if motivational interviewing supports change in modifiable cardiovascular risk factors (smoking status, dietary eating patterns, physical activity levels, lipid profile levels, blood pressure levels, weight, waist circumference, body mass index). |
| Additional outcomes. | The secondary outcome is to identify the requirements for the intervention to be effective. This review will extract data which may be indicators of effectiveness. This will be defined in terms of the reported intervention characteristics: the number, duration, type and setting of sessions, characteristics of the deliverer (professional discipline, training and experience), and the reported motivation interviewing content of the session. Decisions will be based on the methodological quality of the studies. |
| Data extraction. | A standardised form was developed and piloted for data extraction of the eligible studies (J. Higgins, 2011). Key information extracted included:  1. The characteristics of included studies;  • Study design  • Randomisation specifics (cluster/cross-over)  • Duration of follow-up  2. Participant characteristics;  • Total number  • Setting (e.g. hospital, community)  • Diagnostic criteria (e.g. hyperlipidaemia, obesity)  • Age  • Sex  • Country  • Ethnicity  3. Assessments of risk of bias domains;  • Selection bias  • Performance bias  • Detection bias  • Attrition bias  • Reporting bias  4. Nature of intervention;  • Total number of intervention groups  • Intervention specifics (sufficiency for replication, if feasible)  • Session content- 1. Number and length of sessions, type, 2. Characteristics of the clinician who delivered the intervention, 3. Reporting of MI elements  • Comparator group  5. Type of outcomes measured;  • Time points of collection and reporting  • Definition of outcome, unit of measurements used (e.g. physical activity measured in METS)  6. Relevant findings/results;  • Number of participants allocated to each intervention group  • Sample size of each outcome,  • Details about missing participants  • Summary data for each intervention group (effect on modifiable risk factors)  7. Miscellaneous;  • Funding source  • Key conclusions of authors  • Comments from the study authors  Data will be extracted by two independent researchers. Any disagreements will be solved through discussion. Final decisions on the study inclusion will be made by the principal researcher of this review and another researcher, which have examined the full text reports independently for compliance of studies with the eligibility criteria. |
| Risk of bias assessment. | The Cochrane handbook will be used to facilitate judgement of the level of confidence that will be placed on the conclusions drawn. This will be done by two separate researchers, and if no consensus is reached the third researcher will moderate |
| The quality of the studies will be appraised on the following domains. | 1. selection bias,  2. performance bias,  3. detection bias,  4. attrition bias and  5. reporting bias |
| Strategy of data synthesis. | To identify intervention effectiveness, statistical data such as mean group differences between the intervention groups and control groups, will be recorded. An increase in the mean smoking quit attempts, an increase in the mean physical activity level and cardio-protective diet adherence, a decrease in the mean blood pressure level, in serum cholesterol, waist circumference and body mass index will be considered as lifestyle improvements, if statistically significant. Any trends identified across the results will be explored. Whenever quantitative synthesis is not possible, narrative synthesis is planned. Furthermore, in conjunction with the systematic review, when there is sufficient homogeneity, statistical meta-analysis will be performed. To report the meta-analyses results, forest plots, will be used to generate data output. Tests for heterogeneity will be carried out to address whether the variation in the study results is due to genuine heterogeneity differences or homogeneity alone. Random effects meta-analyses will be used as this is more conservative than using fixed effects. If the outcomes are consistent across studies, unstandardized measures to construct meta-analyses will be used. To quantitatively measure the variability between results, the I² statistic will be used. |

**Table S3 Search strategy**

| Literature search for the period 2013 to March week 3 2020 | Searches | | CINAHL Complete (EBSCO) | APA PsycINFO (EBSCO) | Academic Search Ultimate (EBSCO) | Cochrane Central Register of Controlled Trials (EBSCO) | E-Journals (EBSCO) | MEDLINE Complete (EBSCO) | Psychology Database | PubMed | PLOS | DynaMed Plus |
| --- | --- | --- | --- | --- | --- | --- | --- | --- | --- | --- | --- | --- |
| **Concepts** | Search terms; truncations and Boolean operators applied |  |  |  |  |  |  |  |  |  |  |  |
| **Concept 1- Coronary heart disease** | "coronary disease*" OR "cerebrovascular disorder*" OR "cardiovascular disease*" OR "cardiovascular disorder*" OR "cerebrovascular disease*" OR "heart disease*" OR "myocardial infarction" OR "heart disease*" OR "coronary*disease" OR "ischemic*disease" OR "athero*" OR "myocardial" | All fields | 259,914 | 53,869 | 515,344 | 105,059 | 470,881 | 1,094,544 | 121,894 | 766752 |  |  |
|  | **AND** |  |  |  |  |  |  |  |  |  |  |  |
| **Concept 2- Motivational interviewing** | "motivation" OR "motivational interviewing" OR "counseling" OR "counsel*" OR "nondirective therapy" | All fields | 3,331 | 2,179 | 4,158 | 2,093 | 2,360 | 6,827 | 23,649 | 4317 | 307 (search term used; motivational interviewing) | 61 (search term used; motivational interviewing) |
|  | **AND** |  |  |  |  |  |  |  |  |  |  |  |
| **Concept 3- Adult** | "adult*" | All fields | 1,255 | 818 | 678 | 1,004 | 335 | 2,839 | 18,825 | 1655 |  |  |
|  | **AND** |  |  |  |  |  |  |  |  |  |  |  |
| **Concept 4- Prevention** | "prevention" OR "primary prevention" | All fields | 566 | 217 | 225 | 491 | 79 | 1,221 | 12,480 | 708 |  |  |
|  | **AND** |  |  |  |  |  |  |  |  |  |  |  |
| **Concept 5- Risk modification** | "modification of risk" OR "risk modification" OR "compliance" OR "patient compliance" OR "guideline adherence" OR "adherence" OR "lifestyle" OR "behavio#r*" OR "blood pressure" OR "cholesterol" OR "LDL" OR "HDL" OR "dietary" OR "lipids" OR "weight" OR "waist circumference" OR "obesity" OR "body mass index" OR "smoking" OR "smoking cessation" AND ("tobacco" OR " cigarette*") OR "tobacco" OR "tobacco use cessation" OR "physical activity" OR "exercise" OR "exercise tolerance" OR "exercise" OR "exercise capacity" OR "food habits" OR "diet*" AND ("pattern*" OR "habit*" OR "Mediterranean" OR "change*") OR "health* behavio#r" OR "behavio#r" | All fields | 519 | 185 | 205 | 476 | 75 | 1,099 | 11,879 | 121 |  |  |
|  | **AND** |  |  |  |  |  |  |  |  |  |  |  |
| **Concept 6- Clinical trial** | "clinical trial" OR "comparative study" OR "experimental study" OR "controlled trial" OR "random assignment" OR "random allocation" OR "randomized" OR "randomised" OR "experimental group" OR "control group" | All fields | 190 | 49 | 80 | 574 | 26 | 367 | 8,409 | 75 |  |  |
| **After date limiter applied (1980-Mar 2020)** |  | | 184 | 49 | 77 | 571 | 13 | 365 | 3,910 | 28 | 279 | 61 |
| **After selecting peer reviewed journals only** |  | | 177 | 47 | 77 (option not available) | 571 (option not available) | 13 (option not available) | 365 (option not available) | 2,296 | 28 (option not available) |  |  |
| **After excluding children and adolescents** |  | | 155 | 45 | 77 (option not available) | 571 (option not available) | 13 (option not available) | 336 | 403 | 28 (option not available) | 279 | 61 |
| **Total records through database and e-journal searching** |  | | 1968 | | | | | | | | | |
| **Records after duplicates removed** |  | | 1668 | | | | | | | | | |
| **Records after reading titles and abstracts** |  | | 76 | | | | | | | | | |
| **Records after reading studies in full text and eligible criteria applied** |  | | 12 selected papers. 64 excluded, reasons were; 22 ineligible intervention, 4 ineligible design, 20 protocol version, 17 ineligible population, 1 ineligible comparison | | | | | | | | | |
| **Selected studies that fulfilled eligibility criteria** |  | | 12 | | | | | | | | | |

| Records after reading titles and abstracts: |
| --- |
| Reason for exclusion: ineligible intervention |
| 1. Long-term effects of an occupational health guideline on employees' body weight-related outcomes, cardiovascular disease risk factors, and quality of life: results from a randomized controlled trial 2. Effectiveness of a Community Health Worker Cardiovascular Risk Reduction Program in Public Health and Health Care Settings 3. Metabolic risk management, physical exercise and lifestyle counselling in low-active adults: controlled randomized trial (BELLUGAT) 4. Effects of a home-based intervention on diet and physical activity behaviours for rural adults with or at risk of metabolic syndrome: a randomised controlled trial 5. Metabolic syndrome in rural Australia: An opportunity for primary health care 6. Using a smartphone app in changing cardiovascular risk factors: A randomized controlled trial (EVIDENT II study). 7. Development of a peer support intervention to encourage dietary behaviour change towards a Mediterranean diet in adults at high cardiovascular risk. 8. Improving cardiometabolic health through nudging dietary behaviours and physical activity in low SES adults: design of the Supreme Nudge project. 9. The long-term effect of screening and lifestyle counseling on changes in physical activity and diet: The Inter99 study – A randomized controlled trial. 10. The effects of a lifestyle intervention on leisure-time sedentary behaviors in adults at risk: The Hoorn Prevention Study, a randomized controlled trial. 11. Effect of an intervention to improve the cardiovascular health of family members of patients with coronary artery disease: a randomized trial 12. Randomized controlled trial on the long-term efficacy of a multifaceted, interdisciplinary lifestyle intervention in reducing cardiovascular risk and improving lifestyle in patients at risk of cardiovascular disease 13. In overweight or obese patients with diabetes, a lifestyle intervention increased weight loss at 8 years 14. The long-term effect of a population-based life-style intervention on smoking and alcohol consumption. The Inter99 Study-a randomized controlled trial 15. Lifestyle modification and weight reduction among low-income patients with the metabolic syndrome: the CHARMS randomized controlled trial 16. A Randomized-Controlled, Pilot Intervention on Diabetes Prevention and Healthy Lifestyles in the New York City Korean Community 17. Extended and standard duration weight-loss programme referrals for adults in primary care (WRAP): a randomised controlled trial ( 18. Randomized Controlled Trial of E-Counselling for Hypertension: REACH. 19. The motivational interview's impact on inactive diabetic patients' adherence to physical activity: A pilot study of a clinical trial \| [Impacto de la Entrevista Motivacional en la Adherencia de Pacientes Diabéticos Inactivos a la Actividad Física: Estudio Piloto de un Ensayo Clínico emoactif-DM] 20. Comparison of In-Person and Online Motivational Interviewing-Based Health Coaching 21. Landmark Lipid‐Lowering Trials in the Primary Prevention of Cardiovascular Disease 22. Nursing case management for people with hypertension in primary health care: A randomized controlled trial |
| Reason for exclusion: ineligible design |
| 1. Should we give up on motivational interviewing? Not so fast  2. I Move: systematic development of a web-based computer tailored physical activity intervention, based on motivational interviewing and self-determination theory.  3. NHS health checks through general practice: randomised trial of population cardiovascular risk reduction  4. A brief behavioral feedback intervention in hospital outpatients with a high cardiovascular risk |
| Reason for exclusion: Primary/secondary prevention study protocols |
| 1. Comparing the effectiveness of an enhanced MOtiVational intErviewing InTervention (MOVE IT) with usual care for reducing cardiovascular risk in high risk subjects: study protocol for a randomised controlled trial. Trials 2015 2. A 30-month worksite-based lifestyle program to promote cardiovascular health in middle-aged bank employees: Design of the TANSNIP-PESA randomized controlled trial. 3. Design and baseline characteristics of the PerfectFit study: a multicenter cluster-randomized trial of a lifestyle intervention in employees with increased cardiovascular risk. 4. Physical activity prescription by primary care nurses using health assets: Study design of a randomized controlled trial in patients with cardiovascular risk factors 5. Complex multiple risk intervention to promote healthy behaviours in people between 45 to 75 years attended in primary health care (EIRA study): study protocol for a hybrid trial 6. Design and baseline characteristics of the PerfectFit study: a multicenter cluster-randomized trial of a lifestyle intervention in employees with increased cardiovascular risk 7. A 30-month worksite-based lifestyle program to promote cardiovascular health in middle-aged bank employees: Design of the TANSNIP-PESA randomized controlled trial 8. The 40-Something randomized controlled trial to prevent weight gain in mid-age women 9. Development and Testing of a Personalized Web-Based Diet and Physical Activity Intervention Based on Motivational Interviewing and the Self-Determination Theory: Protocol for the MyLifestyleCoach Randomized Controlled Trial 10. Combined use of smartphone and smartband technology in the improvement of lifestyles in the adult population over 65 years: study protocol for a randomized clinical trial (EVIDENT-Age study). 11. Effect of training community health workers and their interventions on cardiovascular disease risk factors among adults in Morogoro, Tanzania: study protocol for a cluster randomized controlled trial. 12. A 30-month worksite-based lifestyle program to promote cardiovascular health in middle-aged bank employees: Design of the TANSNIP-PESA randomized controlled trial. 13. The Trial Using Motivational Interviewing and Positive Affect and Self-Affirmation in African-Americans with Hypertension (TRIUMPH): From theory to clinical trial implementation 14. Design and baseline characteristics of the PerfectFit study: A multicenter cluster-randomized trial of a lifestyle intervention in employees with increased cardiovascular risk 15. Effectiveness of a tailored intervention to improve cardiovascular risk management in primary care: Study protocol for a randomised controlled trial 16. The 40-Something randomized controlled trial to prevent weight gain in mid-age women 17. A 30-month worksite-based lifestyle program to promote cardiovascular health in middle-aged bank employees: Design of the TANSNIP-PESA randomized controlled trial 18. Physical activity prescription by primary care nurses using health assets: Study design of a randomized controlled trial in patients with cardiovascular risk factors 19. A randomised controlled trial of a physical activity and nutrition program targeting middle-aged adults at risk of metabolic syndrome in a disadvantaged rural community health behavior, health promotion and society 20. Effect of motivational interviewing in hypertensive patients (MIdNIgHT): study protocol for a randomized controlled trial |
| Reason for exclusion: ineligible population - Secondary prevention |
| 1. Smartphone-based home care model improved use of cardiac rehabilitation in post myocardial infarction patients: results from a randomised controlled trial FREE 2. Improving Adherence to Secondary Stroke Prevention Strategies Through Motivational Interviewing: Randomized Controlled Trial. 3. Effectiveness of Motivational Interviewing for secondary stroke prevention Reduction in 2-year recurrent risk score and improved behavioral outcomes after participation in the "Beating Heart Problems" self-management program: results of a randomized controlled trial. 4. Telephone-delivered lifestyle support with action planning and motivational interviewing techniques to improve rehabilitation outcomes. 5. Improving Adherence to Secondary Stroke Prevention Strategies Through Motivational Interviewing: Randomized Controlled Trial 6. Enhancing physical activity in cardiac patients who report hopelessness: Feasibility testing of an intervention. 7. Designing and manufacturing of educational multimedia software for preventing coronary artery disease and-its effects on modifying the risk factors in patients with coronary artery disease. 8. Effect of a Telephone-Delivered Coronary Heart Disease Secondary Prevention Program (ProActive Heart) on Quality of Life and Health Behaviours: Primary Outcomes of a Randomised Controlled Trial 9. Optimizing a Positive Psychology Intervention to Promote Health Behaviors After an Acute Coronary Syndrome: The Positive Emotions After Acute Coronary Events III (PEACE-III) Randomized Factorial Trial. 10. Improving Adherence to Secondary Stroke Prevention Strategies Through Motivational Interviewing: Randomized Controlled Trial. 11. Telephone-delivered lifestyle support with action planning and motivational interviewing techniques to improve rehabilitation outcomes 12. The MOTIV-HEART study: A prospective, randomized, single-blind pilot study of brief strategic therapy and motivational interviewing among cardiac rehabilitation patients 13. Patient cardiovascular risk self-management: results from a randomized trial of motivational interviewing delivered by practice nurses 14. A Multicomponent Behavioral Intervention to Reduce Stroke Risk Factor Behaviors The Stroke Health and Risk Education Cluster-Randomized Controlled Trial 15. Reducing recurrent stroke: Methodology of the motivational interviewing in stroke (MIST) randomized clinical trial 16. Investigation of Motivational Interviewing and Prevention Consults to Achieve Cardiovascular Targets (IMPACT) trial. 17. Effectiveness of Nurse Based Motivational Interviewing for Smoking Cessation in High Risk Cardiovascular Outpatients: A Randomized Trial |
| Reason for exclusion: ineligible comparison |
| 1.Functional imagery training versus motivational interviewing for weight loss: a randomised controlled trial of brief individual interventions for overweight and obesity (both interventions include MI) |
| Selected studies that fulfilled eligibility criteria: |
| 1. Effectiveness of the blended-care lifestyle intervention 'PerfectFit': a cluster randomised trial in employees at risk for cardiovascular diseases. 2. Motivational Counselling to Reduce Sitting Time: A Community-Based Randomized Controlled Trial in Adults. 3. Effectiveness of motivational interviewing in patients with dyslipidemia: a randomized cluster trial 4. Worksite weight management program: A three-months intervention study in a primary health care setting 5. Motivational interviewing and problem-solving treatment to reduce type 2 diabetes and cardiovascular disease risk in real life: a randomized controlled trial 6. Reducing weight and increasing physical activity in people at high risk of cardiovascular disease: a randomised controlled trial comparing the effectiveness of enhanced motivational interviewing intervention with usual care 7. Effects of telephone-based motivational interviewing in lifestyle modification program on reducing metabolic risks in middle-aged and older women with metabolic syndrome: A randomized controlled trial. 8. Results from the trial using motivational interviewing, positive affect and self-affirmation in African Americans with hypertension (TRIUMPH). 9. Short and long term effects of a lifestyle intervention for construction workers at risk for cardiovascular disease: a randomized controlled trial 10. Sustained body weight reduction by an individual-based lifestyle intervention for workers in the construction industry at risk for cardiovascular disease: Results of a randomized controlled trial 11. A randomised controlled trial on the effectiveness of a primary health care based counselling intervention on physical activity, diet and CHD risk factors 12. Improving lifestyle and risk perception through patient involvement in nurse-led cardiovascular risk management: A cluster-randomized controlled trial in primary care |

**Table S4 Data extraction sheet.**

| **Characteristics of included studies** | | | | | | | | | | | | | | | | | | | | | |
| --- | --- | --- | --- | --- | --- | --- | --- | --- | --- | --- | --- | --- | --- | --- | --- | --- | --- | --- | --- | --- | --- |
| **Bóveda-Fontán, Barragán-Brun (20)** | | | | | | | | | | | | | | | | | | | | | |
| Methods | | | | | | Multicentre, open, controlled, randomized,  cluster, two-parallel arm trial | | | | | | | | | | | | | | | |
| Participants | | | | | | Patients from 25 community health centres  Unit of randomization:  Inclusion criteria: 40-75 years of age, history of hypercholesterolemia  Exclusion criteria: Secondary causes of dyslipidaemia needing statin agents; Established CVD or chronic diabetes, severe COPD, cancer, hepatic failure, chronic kidney failure, substance and alcohol abuse; individuals who unable to comply with the study procedures or follow-up review due to work; pregnant or nursing mothers; and individuals on anti-diabetic agents.  227 participants randomized: n= 227 | | | | | | | | | | | | | | | |
| Duration of follow-up | | | | | | 12-month follow-up | | | | | | | | | | | | | | | |
| ***Risk of bias*** | | | | | | | | |  | | | | | | | | | | | | |
| **Bias** | | | | | | **Authors’ judgement** | | | **Support for judgement** | | | | | | | | | | | | |
| - Selection bias | | | | | | | | |  | | | | | | | | | | | | |
| Random sequence generation | | | | | | Unclear risk | | | There are no details about the method used to generate the allocation sequence. Quote “We carried out a multicentre, open, controlled, randomized,  Cluster, two-parallel arm trial”. As this detail is insufficient and we cannot say whether the randomization method used, have produced comparable groups.  Therefore, the overall comments for this domain, since no information was provided to permit judgement of ‘low risk’ or ‘high risk’, we judge this domain as ‘unclear risk’ of bias | | | | | | | | | | | | |
| Allocation concealment | | | | | | High risk | | | This study has used an open random schedule, where patients were recruited by their general practitioner. Therefore, general practitioners could possibly foresee assigning of participants and thus introducing selection bias.  Therefore, the overall comments for this domain, since the investigators of this trial did not use a method to conceal allocation such as central allocation or sequentially numbered opaque sealed envelope technique, the judgement of this domain is ‘high risk’ of bias | | | | | | | | | | | | |
| - Performance bias | | | | | | | | |  | | | | | | | | | | | | |
| Blinding of participants and personnel | | | | | | High risk | | | No blinding method was mentioned; therefore, the outcome is likely to be influenced by lack of blinding. We do understand the fact that personnel delivering the intervention cannot be blinded, however there is no detail that ensures that the patients were blinded to the intervention being received.  Therefore, the overall comments for this domain, since no blinding method was described with patients, we judge this domain as being at ‘high risk’ of bias | | | | | | | | | | | | |
| - Detection bias | | | | | | | | |  | | | | | | | | | | | | |
| Blinding of outcome assessment | | | | | | Low risk | | | The study did not address this outcome. However, since all outcome assessments have been measured objectively, this may have not influenced the outcome measurements.  Therefore, the overall comments for this domain, since objective measurements were used, we do believe that this should not introduce bias, therefore the judgment is ‘low risk’ of bias | | | | | | | | | | | | |
| - Attrition bias | | | | | | | | |  | | | | | | | | | | | | |
| Incomplete outcome data | | | | | | Low risk | | | The study has reported the missing outcome data, which is balanced in numbers across both groups, with similar reasons for missing data across groups.  Therefore, the overall comments for this domain is that the attrition bias is of ‘low risk’. | | | | | | | | | | | | |
| - Reporting bias | | | | | | | | |  | | | | | | | | | | | | |
| Selective reporting | | | | | | Low risk | | | A study protocol is available and all the study’s pre-specified outcomes have been reported in the pre-specified way.  Therefore, we judge this domain as being at ‘low risk’ of reporting bias. | | | | | | | | | | | | |
| **3. Participant characteristics;** | | | | | |  | | | | | | | | | | | | | | | |
| Total number | | | | | | 227 | | | | | | | | | | | | | | | |
| Setting (e.g. hospital, community) | | | | | | Community clinics | | | | | | | | | | | | | | | |
| Diagnostic criteria (e.g. hyperlipidaemia, obesity) | | | | | | uncontrolled dyslipidaemia | | | | | | | | | | | | | | | |
| Age | | | | | | 40 to 75 years | | | | | | | | | | | | | | | |
| Sex | | | | | | Men and women | | | | | | | | | | | | | | | |
| Country | | | | | | Spain | | | | | | | | | | | | | | | |
| Ethnicity | | | | | | White Caucasians | | | | | | | | | | | | | | | |
| **4. Nature of intervention;** | | | | | |  | | | | | | | | | | | | | | | |
| Total number of intervention groups | | | | | | 2 groups | | | | | | | | | | | | | | | |
| Intervention specifics (sufficiency for replication, if feasible) | | | | | | MI-based approach with clinical dyslipidaemia protocol recommendations. | | | | | | | | | | | | | | | |
| Session content- | | | | | | | | | | | | | | | | | | | | | |
| number of sessions | | | | | | 5 sessions | | | | | | | | | | | | | | | |
| type | | | | | | In person consultations with  the patients' usual GPs | | | | | | | | | | | | | | | |
| characteristics of the clinician who delivered the intervention | | | | | | General practitioners had training consisting of 16-hour training. Training was delivered by a person with expertise in MI. This focused on the eight basic MI tasks. After completion of the training program. Physicians attended an individual feedback session with an expert in MI. Initial training was reinforced and maintained during work shop through the following actions: a) GP received "educational micro pills" regularly via Internet and SMS messages; b) each GP was assigned a task. Feedback was then received later; c) GPs attended group sessions to evaluate their own performance with real patients using Problem Based Interviewing techniques. | | | | | | | | | | | | | | | |
| Reported intervention elements using TIDieR and MI checklist | | | | | | \| 1. Brief name \| 2. Why \| 3. What materials \| 4. What procedures \| 5. Who provided \| 6. How \| 7. Where \| 8. When and how much \| 9. Tailoring \| 10. Modifications \| 11. Planned strategies to maintain fidelity \| 12. Extent to which intervention was delivered as planned \| \| --- \| --- \| --- \| --- \| --- \| --- \| --- \| --- \| --- \| --- \| --- \| --- \| \| - \| + \| + \| + \| + \| + \| + \| + \| - \| - \| + \| - \| \| 1. Evocation \| 2. Developing a change plan \| 3. compassion \| 4. affirmation \| 5. profound acceptance \| 6. Open-ended questions \| 7. reflection \| 8. Rolling with resistance \| 9. Eliciting and strengthening change talk \| 10. Summarization \| 11. Recognizing and reinforcing change talk \| 12. Consolidating a client's commitment \| \| - \| - \| - \| - \| - \| - \| - \| - \| - \| - \| - \| - \| | | | | | | | | | | | | | | | |
| **5. Type of outcomes measured;** | | | | | |  | | | | | | | | | | | | | | | |
| Time points of collection and reporting | | | | | | At baseline and at 12 months | | | | | | | | | | | | | | | |
| Definition of outcome, unit of measurements used | | | | | | Lipid parameters | | | | | | | | | | | | | | | |
| **6. Relevant findings/results;** | | | | | | Intervention | | | | | | | | Control | | | | | | | |
| Number of participants allocated to each intervention group | | | | | | 107 patients | | | | | | | | 120 patients | | | | | | | |
| Sample size of each outcome, | | | | | | 98 patients | | | | | | | | 98 patients | | | | | | | |
| Details about missing participants | | | | | | 5 patients lost at 2 months (3 refused to continue, 2 did not go back). Another 2 patients lost at 4 months (did not go back). Another 2 lost at 8 months (1 personal problem and the other a serious illness) | | | | | | | | 6 patients lost at 2 months (4 patients did not go back, 1 refused to continue, 1 changed job position). Another 3 lost at 4 months (1 did not go back, 1 changed residence, 1 changed job). 9 patients lost at 8 months (3 did not go back, 6 changed job position) | | | | | | | |
| Lost to follow rate | | | | | | 13.6 % | | | | | | | | | | | | | | | |
| Summary data for each intervention group | | | | | | **Total sample** | | | | | | | | **Estimated between group effect** | | | | | | | |
| Lipid profile | | | | | |  | | | | | | | | | | | | | | | |
| Total cholesterol (mg/dl) | | | | | | MD- = −19.60; 95 % 95 % CI: −15.33 to  −23.87 mg/dl; Friedman test = 91.756; p < 0.001 | | | | | | | | F = 0.021; p = 0.996 | | | | | | | |
| LDL Cholesterol (mg/dl) | | | | | | MD = −13.78; 95 % CI: −9.77 to −17.79 mg/dl; Friedman test =  58.856; p < 0.001 | | | | | | | | F = 0.067; p = 0.977 | | | | | | | |
| Triglycerides (mg/dl) | | | | | | −19.14; 95 % CI: −11.29 to  −26.99 mg/dl; Friedman test = 23.390; p < 0.001 | | | | | | | | F = 0.216; p = 0.886 | | | | | | | |
| Diet status –using MEDITERRANEAN DIET  Questionnaire Score (Mean difference) | | | | | | MD = 1.11; 95 % CI: 1.42-  7.29; Friedman test = 44.366; p < 0.001 | | | | | | | | 95 % CI of MD: −0.626 to 0.582; p >0.05 | | | | | | | |
| Physical activity status- using IPAQ: No. (%) | | | | | | Chi-squared =  23.3; p < 0.01 | | | | | | | | Chi-squared =  23.3; p < 0.01 | | | | | | | |
| Smoking status (%) | | | | | | -33.0 | | | | | | | | n/a | | | | | | | |
| Anthropometric measurement status | | | | | | | | | | | | | |  | | | | | | | |
| Weight | | | | | | −1.77 kg;  95 % CI: −0.91 to −2.64 kg; Friedman = 47.599;  p < 0.001 | | | | | | | | F = 1.258; p = 0.285 | | | | | | | |
| Waist circumference | | | | | | MD = −0.100 to −1.607 cm;  Friedman = 47.086; p < 0.001 | | | | | | | | F = 0.927; p = 0.449 | | | | | | | |
| **7.Miscellaneous;** | | | | | |  | | | | | | | | | | | | | | | |
| Funding source | | | | | | LAPT and JBF conceived the study | | | | | | | | | | | | | | | |
| Key conclusions of authors | | | | | | Both MI and Standard Care significantly reduced total cholesterol, LDL-cholesterol and triglyceride levels, as well improved Mediterranean diet score and weight management after a one-year follow-up program.  MI performed better than usual care in achieving objectives namely lipid control and physical exercise.  It is necessary that quality studies continue research about the use of MI.  Health professionals can learn MI skills to integrate into practice and help achieve changes in patients. There is the possibility to combine it with other educational interventions. | | | | | | | | | | | | | | | |
| Comments from the study authors | | | | | | Nil | | | | | | | | | | | | | | | |
| **The characteristics of included studies;** | | | | | | | | | | | | | | | | | | | | | |
| **Kouwenhoven-Pasmooij, Robroek (21)** | | | | | | | | | | | | | | | | | | | | | |
| Methods | | | | | | | | | Randomized, Cluster randomisation using R version 3.0.1. | | | | | | | | | | | | |
| Participants | | | | | | | | | Participants from occupational health centres  Inclusion criteria:  Exclusion criteria:  Participants randomized: n=491 | | | | | | | | | | | | |
| Duration of follow-up | | | | | | | | | 12 months | | | | | | | | | | | | |
| ***Risk of bias*** | | | | | | | | |  | | | | | | | | | | | | |
| **Bias** | | | | **Authors’ judgement** | | | | | **Support for judgement** | | | | | | | | | | | | |
| - Selection bias | | | |  | | | | | | | | | | | | | | | | | |
| Random sequence generation | | | | Low risk | | | | | A computer random number generator was used, therefore the judgement is of ‘low risk’ of bias towards the sequence generation process | | | | | | | | | | | | |
| Allocation concealment | | | Low risk | | | | | | Randomization was performed by a researcher who was not involved in the trial, therefore allocation was concealed which makes this domain at ‘low risk’ of bias. | | | | | | | | | | | | |
| - Performance bias | | | | | | | | | | | | | | | | | | | | | |
| Blinding of participants and personnel | | High risk | | | | | | | Occupational health physicians, lifestyle coaches and participants were not blinded, however the authors judge that due to the design, it was not possible for blinding. Therefore, we judge bias at ‘high risk’ | | | | | | | | | | | | |
| - Detection bias | | | | | | | | | | | | | | | | | | | | | |
| Blinding of outcome assessment | | High risk | | | | | | | Blinding did not take place and the authors judged that this was due to the nature of the study design. However, we think that this can lead to biased of estimates of the intervention effect. A step which could have been taken to avoid this element of bias, is the use of an independent assessor. Therefore, we judge this domain as having ‘high risk’ of bias. | | | | | | | | | | | | |
| - Attrition bias | | | | | | | | | | | | | | | | | | | | | |
| Incomplete outcome data | | Low risk | | | | | | | We judge that the authors of this study have applied statistical control to imbalanced factors effectively. Non-response analyses were conducted to determine if drop-out was associated with any baseline characteristics or with the sort of intervention used. For Imputation of missing baseline characteristics, the authors have used the mice package in R. Since the percentage of missing values was low the authors have used single imputation.  Missing values of adjustment variables were imputed using chained equations using the mice package in R.  Therefore, we judge this domain as being at ‘low risk’ of bias | | | | | | | | | | | | |
| - Reporting bias | | | | | | | | | | | | | | | | | | | | | |
| Selective reporting | High risk | | | | | | | | A study protocol is available, but not all pre-specified outcomes that are of interest in the review have been reported in the pre-specified way. For this reason, we judge this domain as having a ‘high risk’ of bias | | | | | | | | | | | | |
| **3. Participant characteristics;** | | | | | | | | |  | | | | | | | | | | | | |
| Total number | | | | | | | | | 491 | | | | | | | | | | | | |
| Setting (e.g. hospital, community) | | | | | | | | | occupational health centres | | | | | | | | | | | | |
| Diagnostic criteria (e.g. hyperlipidaemia, obesity) | | | | | | | | | Clients with elevated risk for  CVD | | | | | | | | | | | | |
| Age | | | | | | | | | 40 years and over | | | | | | | | | | | | |
| Sex | | | | | | | | | Both males and females | | | | | | | | | | | | |
| Country | | | | | | | | | Netherlands | | | | | | | | | | | | |
| Ethnicity | | | | | | | | | White Caucasian | | | | | | | | | | | | |
| **4. Nature of intervention;** | | | | | | | | |  | | | | | | | | | | | | |
| Total number of intervention groups | | | | | | | | | 2 groups | | | | | | | | | | | | |
| Intervention specifics (sufficiency for replication, if feasible) | | | | | | | | | The intervention group and control group, received an online risk assessment, including personalized feedback based on the participant’s risk, with suggestions for particular health promotion activities. An electronic newsletter was sent, providing information on the intervention and information on a healthy lifestyle. This was sent via email every 2 to 3 months. The intervention group received an extension with seven in person coaching sessions (3 in person and 4 by telephone) with an Occupational health physician, together with more personalized health promotion activities based on motivational elements, and an additional motivational reference letter. | | | | | | | | | | | | |
| Session content- | | | | | | | | | | | | | | | | | | | | | |
| Planned number of sessions | | | | | | | | | seven individual coaching sessions | | | | | | | | | | | | |
| Delivered | | | | | | | | | 4 MI sessions | | | | | | | | | | | | |
| time | | | | | | | | | 140 minutes in total (mean) | | | | | | | | | | | | |
| type | | | | | | | | | 3 face-to-face and 4 by telephone | | | | | | | | | | | | |
| characteristics of the clinician who delivered the intervention | | | | | | | | | Occupational health physicians who received 3 full days of basic training in MI and also 3 follow-up coaching sessions of 4 hours.  The occupational health physicians have applied a client-centred counselling approach using open questions, supporting, reflecting, and raising ambivalence. The starting point of the counselling was problem feedback by discussing the person’s CVD risks and motivation to change health behaviour. | | | | | | | | | | | | |
| Reported intervention elements using TIDieR and MI checklist | | | | | | | | | \| 1. Brief name \| 2. Why \| 3. What materials \| 4. What procedures \| 5. Who provided \| 6. How \| 7. Where \| 8. When and how much \| 9. Tailoring \| 10. Modifications \| 11. Planned strategies to maintain fidelity \| 12. Extent to which intervention was delivered as planned \| \| --- \| --- \| --- \| --- \| --- \| --- \| --- \| --- \| --- \| --- \| --- \| --- \| \| + \| + \| + \| + \| + \| + \| + \| + \| + \| - \| + \| + \| \| 1. Evocation \| 2. Developing a change plan \| 3. compassion \| 4. affirmation \| 5. profound acceptance \| 6. Open-ended questions \| 7. reflection \| 8. Rolling with resistance \| 9. Eliciting and strengthening change talk \| 10. Summarization \| 11. Recognizing and reinforcing change talk \| 12. Consolidating a client's commitment \| \| + \| + \| + \| - \| + \| + \| + \| - \| - \| - \| - \| - \| | | | | | | | | | | | | |
| **5. Type of outcomes measured;** | | | | | | | | |  | | | | | | | | | | | | |
| Time points of collection and reporting | | | | | | | | | At 6 months and at 12 months | | | | | | | | | | | | |
| Definition of outcome, unit of measurements used | | | | | | | | |  | | | | | | | | | | | | |
| **6. Relevant findings/results;** | | | | | | | | | Intervention | | | | Control | | | | | | | | |
| Number of participants allocated to each intervention group | | | | | | | | | 274 | | | | 217 | | | | | | | | |
| Sample size of each outcome, | | | | | | | | | 271 | | | | 213 | | | | | | | | |
| Details about missing participants | | | | | | | | | 49 participants lost to follow up at 6 months. Another 75 lost to follow up at 12 months. 20 participants discontinued during web-based health risk assessment. 155 participants did not receive the full MI sessions. Details reported about missing participants are personal, work, programme, unknown | | | | 87 participants lost to follow up at 6 months. Another 92 lost to follow up at 12 months. 39 discontinued during web-based health risk assessment. Details reported about missing participants are personal, work, programme, unknown. | | | | | | | | |
| Lost to follow rate | | | | | | | | | 34% | | | | | | | | | | | | |
| Summary data for each intervention group at 12 months follow up | | | | | | | | | Intervention | | | | Control | | | | | | | | |
| Lack of physical activity (%) | | | | | | | | | −50.3 | | | −53.6 | | | | | | p = >0.05 | | | |
| Estimated effect for lack of physical activity (difference)  between intervention groups  (95% CI) | | | | | | | | | −5.6 (− 14.2;5.0) | | | | | | | | | | | | |
| Smoking status | | | | | | | | | 0 % reduction | | | −3.2% reduction | | | | | | p = >0.05 | | | |
| Estimated effect for Smoking (%) (difference)  between intervention groups  (95% CI) | | | | | | | | | 8.6 (−0.1;15.7) | | | | | | | | | | | | |
| Anthropometric measurement status | | | | | | | | | | | | | | | | | |  | | | |
| Body weight | | | | | | | | | − 3.12c (− 4.26; -1.99) | | | 0.17 (−1.44;1.77) | | | | | |  | | | |
| Estimated effect for Body weight (kg), (difference)  between intervention groups  (95% CI) | | | | | | | | | − 2.16 (− 5.49;1.17) | | | | | | | | | | | | |
| BMI (kg/mb),  (mean, 95%CI) | | | | | | | | | − 0.69c (− 1.00; -0.39) | | | 0.24 (− 0.20;0.67) | | | | | | p = <0.05 | | | |
| Estimated effect for BMI (kg/mb),  (mean, 95%CI) (difference)  between intervention groups  (95% CI) | | | | | | | | | −0.81 (− 1.87; 0.26) | | | | | | | | | | | | |
| Mean differences from baseline (intervention group) | | | | | | | | | Weight (kgs)- − 3.12 (− 4.26; -1.99); p <0.05  BMI (kgs/m2)- − 0.69 (− 1.00; -0.39); p <0.05  Lack of physical activity (%) -−50.3; p <0.05  Smoking (%)- | | | | | | | | | | | | |
| Estimated between group effect | | | | | | | | | Weight- (− 2.16; 95%CI -5.49-1.17; p >0.05  BMI- − 0.81; 95% CI -1.87-0.26; p >0.05  Lack of physical  activity (%)- -5.6 (- 14.2;5.0); p >0.05  Smoking (%)-8.6 (−0.1;15.7); p>0.05 | | | | | | | | | | | | |
| **7. Miscellaneous;** | | | | | | | | |  | | | | | | | | | | | | |
| Funding source | | | | | | | | | Financial support was provided by ZonMW (grant number: 208030007).  Additional funding was received from Erasmus Medical Centre (grant number: 2013–13110). | | | | | | | | | | | | |
| Key conclusions of authors | | | | | | | | | Via a website with individualised health assessment and information, body weight improved significantly. MI coaching sessions through a website with tailored care is promising in improving anthropometric measurements of at increased CVD risk individuals. | | | | | | | | | | | | |
| Comments from the study authors | | | | | | | | | Several issues may have reduced the beneficial effects of the extensive intervention; methodological issues, insufficient delivery of the intervention, or ineffectiveness for certain outcomes. The methodological limitation is linked to the cluster design with large cluster-size differences (ranging 1–124). This could have caused under-powering of the study. An associated issue is that a cluster RCT is sensitive to allocation bias. This is noted in the imbalance in gender, age, and education at baseline between the extensive and limited intervention groups. In regards to the delivery of MI, both quantity and quality as provided by OPs need to be considered. 75% of the individuals did not adhere with the full attendance of the 7 planned MI sessions. What is interesting is that BMI decreased statistically significantly, which could suggest that the optimum MI-dose is lower than 7 or, alternatively, that this is determined by personal needs rather than one-size-fits-all. The quality of MI in this study is fairly low. MITI thresholds determined insufficient level of MI compliancy. Since the quality of MI is an important factor in effectiveness of MI, a more detailed exploration of MI-fidelity is needed | | | | | | | | | | | | |
| **The characteristics of included studies;** | | | | | | | | | | | | | | | | | | | | | |
| **Lin, Chiang (25)** | | | | | | | | | | | | | | | | | | | | | |
| Methods | | | | | | | | | | 3 group randomized controlled trial | | | | | | | | | | | |
| Participants | | | | | | | | | | Recruited from an outpatient clinic | | | | | | | | | | | |
| Duration of follow-up | | | | | | | | | | 3 months | | | | | | | | | | | |
| **2. Risk of bias** | | | | | | | | | |  | | | | | | | | | | | |
| **Bias** | | | | | **Authors’ judgement** | | | | | **Support for judgement** | | | | | | | | | | | |
| - Selection bias | | | | | | | | | | | | | | | | | | | | | |
| Random sequence generation | | | | | Low risk | | | | | Investigators have made use of computer-generated random serial numbers. ‘low risk’ of bias | | | | | | | | | | | |
| Allocation concealment | | | | | Low risk | | | | | Opaque sealed envelopes. ‘low-risk of bias’ | | | | | | | | | | | |
| - Performance bias | | | | | | | | | | | | | | | | | | | | | |
| Blinding of participants and personnel | | | | | High risk | | | | | No blinding took place; therefore, the outcome can be influenced by lack of blinding. For this reason, we judge this domain as being open to a ‘high risk’ of bias | | | | | | | | | | | |
| - Detection bias | | | | | | | | | | | | | | | | | | | | | |
| Blinding of outcome assessment | | | | | Low risk | | | | | Outcome measures were collected by a separate research nurse, blinded to the group assignment. Therefore, we judge this as being at ‘low risk’ of bias | | | | | | | | | | | |
| - Attrition bias | | | | | | | | | | | | | | | | | | | | | |
| Incomplete outcome data | | | | | Low risk | | | | | Handling of incomplete outcome data have been imputed using appropriate methods. Here the investigators used intension to treat analysis. Therefore, this fits to the criteria of being at ‘low risk’ of bias | | | | | | | | | | | |
| - Reporting bias | | | | | | | | | | | | | | | | | | | | | |
| Selective reporting | | | | | Low risk | | | | | The study protocol is not available, but it is clear that the published reports include all expected outcomes. Therefore, we judge this domain as being at ‘low risk’ of bias | | | | | | | | | | | |
| **3. Participant characteristics;** | | | | | | | | | |  | | | | | | | | | | | |
| Total number | | | | | | | | | | 115 | | | | | | | | | | | |
| Setting (e.g. hospital, community) | | | | | | | | | | Outpatient clinic | | | | | | | | | | | |
| Diagnostic criteria (e.g. hyperlipidaemia, obesity) | | | | | | | | | | Metabolic syndrome | | | | | | | | | | | |
| Age | | | | | | | | | | 40 and over | | | | | | | | | | | |
| Sex | | | | | | | | | | Women | | | | | | | | | | | |
| Country | | | | | | | | | | Taiwan | | | | | | | | | | | |
| Ethnicity | | | | | | | | | | White Asian | | | | | | | | | | | |
| **4. Nature of intervention;** | | | | | | | | | |  | | | | | | | | | | | |
| Total number of intervention groups | | | | | | | | | | three parallel intervention-group design | | | | | | | | | | | |
| Intervention specifics (sufficiency for replication, if feasible) | | | | | | | | | | Women in the experimental and brief groups each received a single, individual, brief (15–20 min) face-to-face lifestyle modification counselling session and an educational brochure. An extension to this, was a 12-week individualized lifestyle modification program that focused on physical activity promotion by telephone-delivered MI for the intervention group | | | | | | | | | | | |
| Session content- | | | | | | | | | | | | | | | | | | | | | |
| number of sessions | | | | | | | | | | 12 telephone-based MI | | | | | | | | | | | |
| time | | | | | | | | | | 15 – 30 minutes each | | | | | | | | | | | |
| type | | | | | | | | | | The lifestyle modification program began with an individualized education session. Then telephone-based MI for twelve weeks. | | | | | | | | | | | |
| characteristics of the clinician who delivered the intervention | | | | | | | | | | The intervention was delivered by a nurse, who is an expert in MI. The nurse delivered individualized MI for 12 weeks that focused on physical activity promotion. The women were reminded each week with 15–30 min telephone calls to engage in an adequate level of physical activity, and were rewarded for achieving their target physical activity level. At each session, the goal was to encourage women to strengthen their motivation and commitment, to increase their physical activity amount using the brochure timetable, and to address any questions or concerns by telephone. | | | | | | | | | | | |
| Reported intervention elements using TIDieR and MI checklist | | | | | | | | | | \| 1. Brief name \| 2. Why \| 3. What materials \| 4. What procedures \| 5. Who provided \| 6. How \| 7. Where \| 8. When and how much \| 9. Tailoring \| 10. Modifications \| 11. Planned strategies to maintain fidelity \| 12. Extent to which intervention was delivered as planned \| \| --- \| --- \| --- \| --- \| --- \| --- \| --- \| --- \| --- \| --- \| --- \| --- \| \| - \| + \| + \| + \| + \| + \| + \| + \| + \| - \| + \| - \| \| 1. Evocation \| 2. Developing a change plan \| 3. compassion \| 4. affirmation \| 5. profound acceptance \| 6. Open-ended questions \| 7. reflection \| 8. Rolling with resistance \| 9. Eliciting and strengthening change talk \| 10. Summarization \| 11. Recognizing and reinforcing change talk \| 12. Consolidating a client's commitment \| \| + \| - \| + \| + \| - \| - \| - \| - \| - \| - \| - \| - \| | | | | | | | | | | | |
| **5. Type of outcomes measured;** | | | | | | | | | | Physical activity | | | | | | | | | | | |
| Time points of collection and reporting | | | | | | | | | | At 12 weeks | | | | | | | | | | | |
| Definition of outcome, unit of measurements used | | | | | | | | | | (MET-min/week) | | | | | | | | | | | |
| **6. Relevant findings/results;** | | | | | | | | | | Experimental | | | | | Brief | | Usual care | | | | |
| Number of participants allocated to each intervention group | | | | | | | | | | 38 | | | | | 38 | | 39 | | | | |
| Sample size of each outcome, | | | | | | | | | | 38 | | | | | 38 | | 39 | | | | |
| Details about missing participants | | | | | | | | | | 1 Withdrew from study (Went abroad) 3Lost to follow-up (Did not return to the outpatient clinic s) | | | | | 6 Lost to follow-up (Did not return to the outpatient clinic s/ moved to another county) | | 5 Were lost to follow-up (Incomplete telephone number to contact/did not return to the outpatient clinics) | | | | |
| Lost to follow rate | | | | | | | | | | 13% | | | | | | | | | | | |
| Summary data for each intervention group | | | | | | | | | | Experimental G vs. Usual G | | | | | Experimental G vs. Brief G | | | | | | |
| Metabolic syndrome (p-value) [confidence interval] | | | | | | | | | | -0.17 (.003) [-0.29to -0.06] | | | | | -0.17 (.02) [-0.27 to -0.03] | | | | | | |
| Lipid profile | | | | | | | | | |  | | | | |  | | | | | | |
| Elevated triglyceride: >150 mg/dl. (p-value) [confidence interval] | | | | | | | | | | -0.03 (.27) [-0.08 to0.02] | | | | | -0.03 (.31) [-0.08 to 0.02] | | | | | | |
| Physical activity amount, MET-min/week | | | | | | | | | | | | | | | | | | | | | |
| Vigorous-intensity PA (p-value) [confidence interval] | | | | | | | | | | 41.1(.26) [-30.8 to 113] | | | | | 21.1(.41) [-28.5 to70.6] | | | | | | |
| Total physical activity (p-value) [confidence interval] | | | | | | | | | | 846(.01) [173to1519] | | | | | 456(.09) [-157 to1070] | | | | | | |
| Moderate-intensity PA (p-value) [confidence interval] | | | | | | | | | | 337(.02) [43.2to630] | | | | | 206(.08) [-26.2 to 438] | | | | | | |
| Walking (p-value) [confidence interval] | | | | | | | | | | 468(.11) [-105 to1042] | | | | | 271(.36) [-310 to 852] | | | | | | |
| Anthropometric measurement, blood pressure status | | | | | | | | | | | | | | | | | | | | | |
| Central Obesity (p-value) [confidence interval] | | | | | | | | | | -0.23 (.001) [-0.37to -0.09] | | | | | -0.16 (.03) [-0.28 to -0.02] | | | | | | |
| Elevated blood pressure (p-value) [confidence interval] | | | | | | | | | | -0.07 (.09) [-0.15 to 0.01] | | | | | 0.06 (.10) [-0.01 to 0.14] | | | | | | |
| Mean differences from baseline (intervention group) | | | | | | | | | | Central obesity- baseline: 32 (84.2), 12 weeks: 24(63.2); 95% CI- 0.00 to 0.08; p<0.03  MetS n (%)- baseline: 38(100), 12 weeks: 31(81.6), 95%CI- 0.00- 0.08; p <0.01  Numbers of MetS risks, mean (SD)-baseline: 4.0 (0.8), 12 weeks: 3.6(1.1), 95% CI- 0.1-0.5; p<0.002 | | | | | | | | | | | |
| Estimated between group effect | | | | | | | | | | Numbers of MetS risks- 0.4 beta; p<0.02 | | | | | | | | | | | |
| **7. Miscellaneous;** | | | | | | | | | |  | | | | | | | | | | | |
| Funding source | | | | | | | | | | This study was funded by the Tri-Service  General Hospital (TSGH-C102-138), Taipei, Taiwan | | | | | | | | | | | |
| Key conclusions of authors | | | | | | | | | | A nurse-delivered individualized lifestyle modification Program, which focused on physical activity promotion using telephone MI sessions, to support middle-aged and older females with metabolic syndrome achieve physical activity targets, reduce metabolic risks, and metabolic syndrome diagnoses. Based on the outcome results, this is an ideal intervention to be used by clinicians to share decision-making with clients based on evidence-based nursing practice. | | | | | | | | | | | |
| Comments from the study authors | | | | | | | | | | Further studies are needed to evaluate long term follow-up effects of the 12-week personalized lifestyle modification program focused on physical activity promotion using MI among middle aged and older adults of both gender having a diagnosis of metabolic syndrome or have metabolic risk/s. Since healthy diet patterns and increased physical activity have different effects on body composition, with both contributing to fat loss, further study might be needed to incorporate diet-pattern modification into future health promotion programs or to comprehensively assess the diet for changes. Moreover, exploratory studies are needed to assess whether the program actually did empower participants. Mixed designs using focus groups or qualitative examination of what participants perceived as most helpful are considered. | | | | | | | | | | | |
| **The characteristics of included studies;** | | | | | | | | | | | | | | | | | | | | | |
| **Aadahl, Linneberg (23)** | | | | | | | | | | | | | | | | | | | | | |
| Method | | | | | | | | | | Randomized, open-end randomized, controlled trial | | | | | | | | | | | |
| Participants | | | | | | | | | | Inclusion criteria: Individuals aged between 18 and 69 years, who self-reported 3.5 hours of daily  leisure-time sedentary behaviours  Exclusion criteria; n/a  Participants randomized: n=166 | | | | | | | | | | | |
| Duration of follow-up | | | | | | | | | | 6 months | | | | | | | | | | | |
| **2. Risk of bias** | | | | | | | **Authors’ judgement** | | | **Support for judgement** | | | | | | | | | | | |
| - Selection bias | | | | | | |  | | | | | | | | | | | | | | |
| Random sequence generation | | | | | | | Low risk | | | open-ended randomization using  Computer-generated random numbers operated by a blinded data manager. | | | | | | | | | | | |
| Allocation concealment | | | | | | |  | | |  | | | | | | | | | | | |
| - Performance bias | | | | | | |  | | | | | | | | | | | | | | |
| Blinding of participants and personnel | | | | | | | Low risk | | | In view this was an open trial where participants and some of the researchers were aware of the randomization, the authors of this study claim that the data of the gadget used were not shown to the participants. Also, the investigators who processed the data of the ActivPAL, were “blinded”. An important step which was taken is that research staff who were directly involved in conducting the objective measurements and data processing, were blinded to the randomization. Therefore, by this information we judge that this domain is at ‘low risk’ of bias. | | | | | | | | | | | |
| - Detection bias | | | | | | |  | | | | | | | | | | | | | | |
| Blinding of outcome assessment | | | | | | | Low risk | | | The investigators who processed the data of the ActivPAL, were “blinded”. An important step which was taken is that research staff who were directly involved in conducting the objective measurements and data processing, were blinded to the randomization. Therefore, we judge this as being at ‘low risk’ of bias | | | | | | | | | | | |
| - Attrition bias | | | | | | |  | | | | | | | | | | | | | | |
| Incomplete outcome data | | | | | | | High risk | | | There is imbalance in numbers of missing outcome data across the intervention groups (control n=5, intervention n=12) and not having similar reasons for missing data across the groups, hence we judge this as being at “high risk” of bias | | | | | | | | | | | |
| - Reporting bias | | | | | | |  | | | | | | | | | | | | | | |
| Selective reporting | | | | | | | Unclear risk | | | There is insufficient information to permit judgement of ‘low risk’ or ‘high risk’. Therefore, the criteria for the judgement is of ‘unclear risk’ of bias | | | | | | | | | | | |
| **3. Participant characteristics;** | | | | | | | | | |  | | | | | | | | | | | |
| Total number | | | | | | | | | | 166 | | | | | | | | | | | |
| Setting (e.g. hospital, community) | | | | | | | | | | community-based trial | | | | | | | | | | | |
| Diagnostic criteria (e.g. hyperlipidaemia, obesity) | | | | | | | | | | sedentary adults | | | | | | | | | | | |
| Age | | | | | | | | | | 18-69 | | | | | | | | | | | |
| Sex | | | | | | | | | | Mixed | | | | | | | | | | | |
| Country | | | | | | | | | | Denmark | | | | | | | | | | | |
| Ethnicity | | | | | | | | | | White Caucasian | | | | | | | | | | | |
| **4. Nature of intervention;** | | | | | | | | | |  | | | | | | | | | | | |
| Total number of intervention groups | | | | | | | | | | 2 group | | | | | | | | | | | |
| Intervention specifics (sufficiency for replication, if feasible) | | | | | | | | | | Intervention using behavioural choice theory, incorporating individual behaviour goal-setting, self-efficacy, and MI techniques. At each of the following sessions (Sessions 2–4), behaviour goals were reviewed and evaluated. With the support of a research nurse, goals were modified and new goals were set. The intervention program focused on four key messages or themes: (1) reduce daily TV viewing; (2) substitute sitting with standing when possible—at work and at home (no time restrictions); (3) break up prolonged sitting by standing up frequently; and (4) 30 minutes maximum of sitting per episode. Written information with key messages, strategies and suggestions for reduction of sitting time, were distributed to participants at each session. | | | | | | | | | | | |
| Session content- | | | | | | | | | | | | | | | | | | | | | |
| number of sessions | | | | | | | | | | 4 sessions | | | | | | | | | | | |
| Time | | | | | | | | | | 30 and 45 minutes each. | | | | | | | | | | | |
| type | | | | | | | | | | individualized face-to-face motivational counselling intervention | | | | | | | | | | | |
| characteristics of the clinician who delivered the intervention | | | | | | | | | | Research nurse. No other details given in regard to the experience or training the research nurse has. | | | | | | | | | | | |
| Reported intervention elements using TIDieR and MI checklist | | | | | | | | | | \| 1. Brief name \| 2. Why \| 3. What materials \| 4. What procedures \| 5. Who provided \| 6. How \| 7. Where \| 8. When and how much \| 9. Tailoring \| 10. Modifications \| 11. Planned strategies to maintain fidelity \| 12. Extent to which intervention was delivered as planned \| \| --- \| --- \| --- \| --- \| --- \| --- \| --- \| --- \| --- \| --- \| --- \| --- \| \| - \| + \| + \| + \| + \| + \| + \| + \| - \| - \| - \| - \| \| 1. Evocation \| 2. Developing a change plan \| 3. compassion \| 4. affirmation \| 5. profound acceptance \| 6. Open-ended questions \| 7. reflection \| 8. Rolling with resistance \| 9. Eliciting and strengthening change talk \| 10. Summarization \| 11. Recognizing and reinforcing change talk \| 12. Consolidating a client's commitment \| \| - \| + \| - \| - \| - \| - \| - \| - \| - \| - \| - \| - \| | | | | | | | | | | | |
| **5. Type of outcomes measured;** | | | | | | | | | |  | | | | | | | | | | | |
| Time points of collection and reporting | | | | | | | | | |  | | | | | | | | | | | |
| Definition of outcome, unit of measurements used | | | | | | | | | | Objectively measured overall sitting time | | | | | | | | | | | |
| **6. Relevant findings/results;** | | | | | | | | | | Intervention | | | | | | Control | | | | | |
| Number of participants allocated to each intervention group | | | | | | | | | | 93 | | | | | | 73 | | | | | |
| Sample size of each outcome, | | | | | | | | | | 93 patients | | | | | | 73 patients | | | | | |
| Details about missing participants | | | | | | | | | | Drop out, n=12  Pregnancy, n=1  Lack of time, n=3  Illness in family, n=2  Unknown reason, n=6 | | | | | | Drop out, n=5  Cancer, n=1  Lack of time, n=1  Unknown reason, n=3 | | | | | |
| Lost to follow rate | | | | | | | | | | 10 % | | | | | | | | | | | |
| Difference in change between groups (95% CI) | | | | | | | | | | | | | | | | | | | | | |
| Lipid profile | | | | | | | | | |  | | | | | | | | | | p-value | |
| Total cholesterol (mg/dl) | | | | | | | | | | -0.18 (-0.39, 0.31) | | | | | | | | | | 0.09 | |
| LDL Cholesterol (mg/dl) | | | | | | | | | | -0.15 (-0.33, 0.04) | | | | | | | | | | 0.11 | |
| Triglycerides (mg/dl) | | | | | | | | | | -0.06 (-0.23, 0.10) | | | | | | | | | | 0.43 | |
| Anthropometric measurement status | | | | | | | | | | | | | | | | | | | |  | |
| Body fat | | | | | | | | | | -0.74 (-1.55, 0.07) | | | | | | | | | | 0.08 | |
| Weight | | | | | | | | | | -0.83 (-1.73, 0.06) | | | | | | | | | | 0.07 | |
| Waist circumference | | | | | | | | | | -1.42 (-2.54, -0.29) | | | | | | | | | | 0.01 | |
| Physical activity status | | | | | | | | | |  | | | | | | | | | |  | |
| Sitting time | | | | | | | | | | -0.32 (-0.87, 0.24) | | | | | | | | | | 0.26 | |
| Standing time | | | | | | | | | | 0.44 (0.8, 0.80) | | | | | | | | | | 0.02 | |
| Stepping time | | | | | | | | | | 0.15 (-0.04, 0.33) | | | | | | | | | | 0.11 | |
| **7. Miscellaneous;** | | | | | | | | | |  | | | | | | | | | | | |
| Funding source | | | | | | | | | | Supported by health insurance foundation & Lundbeck foundation | | | | | | | | | | | |
| Key conclusions of authors | | | | | | | | | |  | | | | | | | | | | | |
| Comments from the study authors | | | | | | | | | | Nil | | | | | | | | | | | |
|  | | | | | | | | | |  | | | | | | | | | | | |
| **Characteristics of included studies** | | | | | | | | | | | | | | | | | | | | | |
| **Lakerveld, Bot (22)** | | | | | | | | | | | | | | | | | | | | | |
| Methods | | | | | | | | parallel group randomized controlled trial | | | | | | | | | | | | | |
| Participants | | | | | | | | Patients from 12 general practices.  Unit of randomization: primary care.  Inclusion criteria: participants with at least a 10.0% T2DM risk and/or CVD mortality risk and no known prevalent T2DM or CVD  Exclusion criteria: Not specified  622 participants randomized: n= 314 intervention, n= 308 control.  Mean (SD) age: 43.6 (5.1) years, females= 56.7% | | | | | | | | | | | | | |
| Duration of follow-up | | | | | | | | 12-month follow-up | | | | | | | | | | | | | |
| ***Risk of bias*** | | | | | | | | | | |  | | | | | | | | | | |
| **Bias** | | | | | | | | **Authors’ judgement** | | | **Support for judgement** | | | | | | | | | | |
| - Selection bias | | | | | | | | | | |  | | | | | | | | | | |
| Random sequence generation | | | | | | | | Low risk | | | A randomization schedule was drawn up using a computerized  random number generator.  Note: Members from the same family were randomized to the same group as the first member, to avoid contamination. | | | | | | | | | | |
| Allocation concealment | | | | | | | | Low risk | | | To ensure concealment of the treatment allocation, an independent individual, who had no information about the study participants, performed the randomization. | | | | | | | | | | |
| - Performance bias | | | | | | | | | | |  | | | | | | | | | | |
| Blinding of participants and personnel | | | | | | | | Unclear risk | | | The research assistants, the principal investigator and the general practitioners were blinded to group assignment. However, the study gives insufficient information how the blinding procedures did take place. Also, it seems that the participants were not blinded. | | | | | | | | | | |
| - Detection bias | | | | | | | | | | |  | | | | | | | | | | |
| Blinding of outcome assessment | | | | | | | | Unclear risk | | | There is insufficient information to permit judgement of ‘low risk or high risk’ | | | | | | | | | | |
| - Attrition bias | | | | | | | | | | |  | | | | | | | | | | |
| Incomplete outcome data | | | | | | | | Low risk | | | Few participants were lost to follow-up (19%). Missing outcome data is balanced in numbers across intervention groups, with similar reasons for missing data across groups | | | | | | | | | | |
| - Reporting bias | | | | | | | | | | |  | | | | | | | | | | |
| Selective reporting | | | | | | | | Unclear risk | | | The authors of the study state that the study protocol was approved by the Medical Ethics Committee of the VU University Medical Centre in Amsterdam, however it seems that this was not published | | | | | | | | | | |
| **3. Participant characteristics;** | | | | | | | |  | | | | | | | | | | | | | |
| Total number | | | | | | | | 622 | | | | | | | | | | | | | |
| Setting (e.g. hospital, community) | | | | | | | | Diabetes Research Centre | | | | | | | | | | | | | |
| Diagnostic criteria (e.g. hyperlipidaemia, obesity) | | | | | | | | High risk profiles for CVD/type 2 diabetes | | | | | | | | | | | | | |
| Age | | | | | | | | 43.5 years (SD 5.3) | | | | | | | | | | | | | |
| Sex | | | | | | | | 363 participants were female (58.4%) | | | | | | | | | | | | | |
| Country | | | | | | | | Netherlands | | | | | | | | | | | | | |
| Ethnicity | | | | | | | | White | | | | | | | | | | | | | |
| **4. Nature of intervention;** | | | | | | | |  | | | | | | | | | | | | | |
| Total number of intervention groups | | | | | | | | 2 groups | | | | | | | | | | | | | |
| Intervention specifics (sufficiency for replication, if feasible) | | | | | | | | Intervention: Trained practice nurses delivered six in person 30-minute counselling sessions, followed by 3-monthly telephone sessions, MI and problem-solving treatment were used. The aim of MI was to make the attitude and intention to change stronger, guided by the Theory of Planned Behaviour. Focusing on discrepancy between the personal goals of the participants and their actual situation was the key method used, as described in the theory of self-regulation. Problem Solving Treatment was used to support participants in finding solutions to overcome this discrepancy, to strengthen their perceived control, and to enable capability to overcome barriers that could stop them from lifestyle modification.  Control: control group received existing leaflets containing health guidelines regarding physical activity and a healthy diet. Smokers received an additional leaflet about smoking cessation. | | | | | | | | | | | | | |
| Session content- | | | | | | | | | | | | | | | | | | | | | |
| number of sessions | | | | | | | | Six followed by 4 telephone sessions | | | | | | | | | | | | | |
| type | | | | | | | | Face to face of 30 minute each | | | | | | | | | | | | | |
| characteristics of the clinician who delivered the intervention | | | | | | | | Practice nurses who received 18 hours of specific training from MI experienced psychologists prior to the intervention (12 hours of MI and 6 hours of Problem-Solving Treatment). During training practice nurses used a treatment manual developed by the project leader and the psychologists who provided the training. Practical coaching was provided halfway through the sessions, and consisted of one hour of individual coaching with feedback. A random selection of sessions per practice nurse, was voice recorded, and used during training. | | | | | | | | | | | | | |
| Reported intervention elements using TIDieR and MI checklist | | | | | | | | \| 1. Brief name \| 2. Why \| 3. What materials \| 4. What procedures \| 5. Who provided \| 6. How \| 7. Where \| 8. When and how much \| 9. Tailoring \| 10. Modifications \| 11. Planned strategies to maintain fidelity \| 12. Extent to which intervention was delivered as planned \| \| --- \| --- \| --- \| --- \| --- \| --- \| --- \| --- \| --- \| --- \| --- \| --- \| \| - \| + \| + \| + \| + \| + \| + \| + \| - \| - \| + \| - \| \| 1. Evocation \| 2. Developing a change plan \| 3. compassion \| 4. affirmation \| 5. profound acceptance \| 6. Open-ended questions \| 7. reflection \| 8. Rolling with resistance \| 9. Eliciting and strengthening change talk \| 10. Summarization \| 11. Recognizing and reinforcing change talk \| 12. Consolidating a client's commitment \| \| + \| - \| - \| - \| - \| - \| - \| - \| - \| - \| - \| - \| | | | | | | | | | | | | | |
| **5. Type of outcomes measured;** | | | | | | | | Primary outcome measures were the estimated risk of developing T2DM (and the estimated risk of CVD mortality (SCORE).  Secondary outcome measures:  Self-reported physical activity using METS  Fruit and vegetable intake using the 8-item food frequency questionnaire  Smoking status using WHO assessment | | | | | | | | | | | | | |
| Time points of collection and reporting | | | | | | | | 6 months, 12 months | | | | | | | | | | | | | |
| Definition of outcome, unit of measurements used | | | | | | | |  | | | | | | | | | | | | | |
| **6. Relevant findings/results;** | | | | | | | | Intervention | | | | | | | | | | | Control | | |
| Number of participants allocated to each intervention group | | | | | | | | Patients  n=249 | | | | | | | | | | | Patients  n=253 | | |
| Sample size of each outcome, | | | | | | | | patients | | | | | | | | | | | patients | | |
| Details about missing participants | | | | | | | | 38 unable to attend  8 withdrew consent  1 became pregnant  9 unable to attend  4 withdrew consent  1 unable to contact  4 had diagnosed  T2DM at follow-up 1  N=65 | | | | | | | | | | | 29 unable to attend  5 withdrew consent  3 became pregnant  2 unable to contact  8 unable to attend  3 withdrew consent  3 unable to contact  1 became pregnant  1 died of CVD  N=55 | | |
| Lost to follow rate | | | | | | | | 19% | | | | | | | | | | | | | |
| Summary data for each intervention group | | | | | | | | Intervention | | | | | | | | | | | Control | | |
| Lipid profile | | | | | | | |  | | | | | | | | | | |  | | p-value |
| Total cholesterol (mg/dl) | | | | | | | | n/a | | | | | | | | | | |  | |  |
| LDL Cholesterol (mg/dl) | | | | | | | | n/a | | | | | | | | | | |  | |  |
| Triglycerides (mg/dl) | | | | | | | | n/a | | | | | | | | | | |  | |  |
| Meeting recommendations fruit intake (%) | | | | | | | | 58 (18.5) | | | | | | | | | | | 68 (22.1) | |  |
| Meeting recommendations vegetable intake | | | | | | | | 62 (19.7) | | | | | | | | | | | 56 (18.2) | |  |
| Self-reported physical activity (METs) | | | | | | | | | | | | | | | | | | | | |  |
| Low or inactive | | | | | | | |  | | | | | | | | | | |  | |  |
| Meeting recommendations (%) | | | | | | | | 162 (51.6) | | | | | | | | | | | 160 (51.9) | |  |
| Smoking status (%) | | | | | | | | 46 (18.3) | | | | | | | | | | | 43 (17) | | n/a |
| **7. Miscellaneous;** | | | | | | | |  | | | | | | | | | | | | | |
| Funding source | | | | | | | | No information reported | | | | | | | | | | | | | |
| Key conclusions of authors | | | | | | | | The provision of this primary prevention approach was not effective in a Dutch ‘real life’ primary care setting. | | | | | | | | | | | | | |
| Comments from the study authors | | | | | | | | Attendance rate of sessions was low, down to 2 counselling sessions. This may have contributed to the absence of an intervention effect. Analysis of the participants who had attended at least 4 counselling sessions showed no improvement. This could mean that 6 or less sessions may not have been enough to induce change. However, as the participants in our effectiveness study were not keen enough to attend 6 sessions, it is unlikely that they would want to attend for more. | | | | | | | | | | | | | |
| **Characteristics of included studies** | | | | | | | | | | | | | | | | | | | | | |
| **Boutin-Foster, Offidani (27)** | | | | | | | | | | | | | | | | | | | | | |
| Methods | | | | | | | | Randomization in a 1:1 ratio to either an active intervention group or an education- based control group. | | | | | | | | | | | | | |
| Participants | | | | | | | | Patients from health centres and community ambulatory practices.  Unit of randomization: primary care.  Inclusion criteria: participants having a diagnosis of  hypertension, being on at least one antihypertensive medication and having an elevated blood pressure reading at the time of recruitment, African American or Black.  Exclusion criteria: Not specified  238 participants randomized: n= 116 intervention, n= 122 control.  Mean (SD) age: 56 (11) years, females= 70% | | | | | | | | | | | | | |
| Duration of follow-up | | | | | | | | 12-month follow-up | | | | | | | | | | | | | |
| ***Risk of bias*** | | | | | | | | | | |  | | | | | | | | | | |
| **Bias** | | | | | | | | **Authors’ judgement** | | | **Support for judgement** | | | | | | | | | | |
| - Selection bias | | | | | | | | | | |  | | | | | | | | | | |
| Random sequence generation | | | | | | | | Unclear risk | | | Equal randomisation 1:1 ratio was used to support a balanced allocation ratio and not to reduce the power of the study, however there is insufficient information how this was done such using a computer-generated randomisation schedule. | | | | | | | | | | |
| Allocation concealment | | | | | | | | High risk | | | Same study personnel administered the follow-up calls to  maintain continuity and therefore were not blinded to randomization  group and any protocol-specific issues. | | | | | | | | | | |
| - Performance bias | | | | | | | | | | |  | | | | | | | | | | |
| Blinding of participants and personnel | | | | | | | | High risk | | | Given the interactive nature of the intervention, participants and assessors cannot be blinded to the study intervention. | | | | | | | | | | |
| - Detection bias | | | | | | | | | | |  | | | | | | | | | | |
| Blinding of outcome assessment | | | | | | | | Unclear risk | | | The study did not address this outcome | | | | | | | | | | |
| Attrition bias | | | | | | | | | | |  | | | | | | | | | | |
| Incomplete outcome data | | | | | | | | Low risk | | | Lost to follow-up (26%). Missing outcome data is balanced in numbers across intervention groups, with similar reasons for missing data across groups | | | | | | | | | | |
| - Reporting bias | | | | | | | | | | |  | | | | | | | | | | |
| Selective reporting | | | | | | | | Unclear risk | | | There is insufficient information to permit judgement, and no protocol was located for this study. | | | | | | | | | | |
| **3. Participant characteristics;** | | | | | | | |  | | | | | | | | | | | | | |
| Total number | | | | | | | | 238 | | | | | | | | | | | | | |
| Setting (e.g. hospital, community) | | | | | | | | health centres and community ambulatory practices | | | | | | | | | | | | | |
| Diagnostic criteria (e.g. hyperlipidaemia, obesity) | | | | | | | | Hypertension | | | | | | | | | | | | | |
| Age | | | | | | | | 56 years (SD 11) | | | | | | | | | | | | | |
| Sex | | | | | | | | 167 participants were female (70%) | | | | | | | | | | | | | |
| Country | | | | | | | | America | | | | | | | | | | | | | |
| Ethnicity | | | | | | | | Black African Americans | | | | | | | | | | | | | |
| **4. Nature of intervention;** | | | | | | | |  | | | | | | | | | | | | | |
| Total number of intervention groups | | | | | | | | 2 groups | | | | | | | | | | | | | |
| Intervention specifics (sufficiency for replication, if feasible) | | | | | | | | Intervention: Research assistants delivered MI-based counselling. The MI intervention included of assessing the motivation and confidence for medication adherence; assessing for barriers which could hinder adherence; eliciting the advantages and disadvantages of any concerns; and a reassessment of their goals and values and linking their current health behaviour pattern to these inner values and life target goals. These strategies were reinforced every 2 months via telephone calls. Participants were provided with an educational workbook plus a positive effect and self-affirmation induction protocol. This helped to focus on positive thoughts that made them feel good throughout their day or when they encountered stressful situations. Moreover, participants had access to a hypertension workbook about the aetiology of blood pressure, treatment options, and lifestyle changes that one could take to modify blood pressure. A behaviour contract was also part of the intervention, where participants were asked to sign this contract that specified steps that they would take in an effort to improve their ability to take their blood pressure medications as prescribed  Control: control group received existing hypertension workbook. Were also asked to develop a behaviour contract that specified steps that they would take in an effort to improve their ability to take their blood pressure medications as prescribed. Received reinforcement every 2 months via telephone calls. | | | | | | | | | | | | | |
| Session content- | | | | | | | | | | | | | | | | | | | | | |
| number of sessions | | | | | | | | One MI session followed by 6 telephone calls | | | | | | | | | | | | | |
| type | | | | | | | | Telephone based | | | | | | | | | | | | | |
| characteristics of the clinician who delivered the intervention | | | | | | | | Research assistants were trained to deliver the intervention components. However, no information was given about what sort of training did they receive and for how long. | | | | | | | | | | | | | |
| Reported intervention elements using TIDieR and MI checklist | | | | | | | | \| 1. Brief name \| 2. Why \| 3. What materials \| 4. What procedures \| 5. Who provided \| 6. How \| 7. Where \| 8. When and how much \| 9. Tailoring \| 10. Modifications \| 11. Planned strategies to maintain fidelity \| 12. Extent to which intervention was delivered as planned \| \| --- \| --- \| --- \| --- \| --- \| --- \| --- \| --- \| --- \| --- \| --- \| --- \| \| + \| + \| + \| + \| + \| + \| + \| + \| - \| - \| - \| - \| \| 1. Evocation \| 2. Developing a change plan \| 3. compassion \| 4. affirmation \| 5. profound acceptance \| 6. Open-ended questions \| 7. reflection \| 8. Rolling with resistance \| 9. Eliciting and strengthening change talk \| 10. Summarization \| 11. Recognizing and reinforcing change talk \| 12. Consolidating a client's commitment \| \| + \| + \| - \| + \| - \| - \| - \| + \| - \| - \| - \| - \| | | | | | | | | | | | | | |
| **5. Type of outcomes measured;** | | | | | | | |  | | | | | | | | | | | | | |
| Time points of collection and reporting | | | | | | | | 12 months | | | | | | | | | | | | | |
| Definition of outcome, unit of measurements used | | | | | | | | Blood pressure control, mmHg | | | | | | | | | | | | | |
| **6. Relevant findings/results;** | | | | | | | | Intervention | | | | | | | | | | | Control | | |
| Number of participants allocated to each intervention group | | | | | | | | Patients  n=116 | | | | | | | | | | | Patients  n=122 | | |
| Sample size of each outcome, | | | | | | | | patients n=90 | | | | | | | | | | | Patients n=87 | | |
| Details about missing participants | | | | | | | | Attrition n=26  Withdrew n=5  Lost contact n=14  Moved n=1  Could not make time commitment n=3  No show to closeout n=2  Death n=1 | | | | | | | | | | | Attrition n=35  Withdrew n=6  Lost contact n=19  Moved n=2  Could not make time commitment n=3  No show to closeout n=3  Death n=1  Travelled out of country n=1 | | |
| Lost to follow rate | | | | | | | | 26 % | | | | | | | | | | | | | |
| Summary data for each intervention group | | | | | | | | Intervention | | | | | | | | | | | Control | | |
|  | | | | | | | |  | | | | | | | | | | |  | | p-value |
| Meeting blood pressure target range of <140/90 | | | | | | | | 83.7% | | | | | | | | | | | 82.2% | | P=.50 |
| **7. Miscellaneous;** | | | | | | | |  | | | | | | | | | | | | | |
| Funding source | | | | | | | | Centre of Excellence in Health Disparities  Research and Community Engagement  (CEDREC) NIMHD P60 MD003421-02. | | | | | | | | | | | | | |
| Key conclusions of authors | | | | | | | | The results illustrate the importance of addressing the psychosocial context of blood pressure medication adherence and blood pressure control.  We also achieved success in 82% of the patients in this setting, which is above the national average of approximately 50% and which is much higher than rates for community health settings. | | | | | | | | | | | | | |
| Comments from the study authors | | | | | | | | While these findings did not demonstrate a difference in control between the two arms, lessons learned can be used to refine behavioural interventions for blood pressure control. | | | | | | | | | | | | | |
| **Characteristics of included studies** | | | | | | | | | | | | | | | | | | | | | |
| **Kong, Jok (26)** | | | | | | | | | | | | | | | | | | | | | |
| Methods | | | | | | | | a single-centre, randomized controlled trial | | | | | | | | | | | | | |
| Participants | | | | | | | | Employees from Sarawak Government Polyclinic.  Unit of randomization: primary care.  Inclusion criteria: aged 18 to 59 years with BMI at  least 18.5 kg/m2 or above, able to understand Bahasa Malaysia or English; not planning to transfer or leave employment in the next 12 months; not pregnant; Malaysian; and not taking any weight loss supplementation three months before beginning of the intervention.  Exclusion criteria: known coronary artery disease or a procedure to treat such disease  143 participants randomized: n=70 intervention, n=70 control.  Mean (SD) age: 34(9) years, females= 72% | | | | | | | | | | | | | |
| Duration of follow-up | | | | | | | | 3-month follow-up | | | | | | | | | | | | | |
| ***Risk of bias*** | | | | | | | | | | |  | | | | | | | | | | |
| **Bias** | | | | | | | | **Authors’ judgement** | | | **Support for judgement** | | | | | | | | | | |
| - Selection bias | | | | | | | | | | |  | | | | | | | | | | |
| Random sequence generation | | | | | | | | High risk | | | The study states ’70 participants (employees), who met the initial screening criteria were recruited into the intervention group. On the other hand, 73 participants from outpatient clinic by physician were recruited as the control group’. Therefore, it seems that allocation was done in a method of non-random categorization of participants. | | | | | | | | | | |
| Allocation concealment | | | | | | | | Unclear risk | | | Insufficient information to permit judgement of low risk or high risk | | | | | | | | | | |
| - Performance bias | | | | | | | | | | |  | | | | | | | | | | |
| Blinding of participants and personnel | | | | | | | | Low risk | | | The authors of the study state ‘Blinding of subjects was performed to avoid unnecessary  interpersonal and intrapersonal bias’. This was done by having different investigators for both groups. Also having the interventions running concurrently at a different treatment room during the 12-week study, so there was no contact between participants during the study. | | | | | | | | | | |
| - Detection bias | | | | | | | | | | |  | | | | | | | | | | |
| Blinding of outcome assessment | | | | | | | | Unclear risk | | | The study did not address this outcome | | | | | | | | | | |
| - Attrition bias | | | | | | | | | | |  | | | | | | | | | | |
| Incomplete outcome data | | | | | | | | Unclear risk | | | This study did not address this outcome | | | | | | | | | | |
| - Reporting bias | | | | | | | | | | |  | | | | | | | | | | |
| Selective reporting | | | | | | | | Unclear risk | | | Insufficient information to permit judgement of ‘low risk’ or ‘high risk’ | | | | | | | | | | |
| **3. Participant characteristics;** | | | | | | | |  | | | | | | | | | | | | | |
| Total number | | | | | | | | 88 | | | | | | | | | | | | | |
| Setting (e.g. hospital, community) | | | | | | | | Community clinic | | | | | | | | | | | | | |
| Diagnostic criteria (e.g. hyperlipidaemia, obesity) | | | | | | | | BMI of 18.5 kg/m2 or above | | | | | | | | | | | | | |
| Age | | | | | | | | 34 years (SD 9) | | | | | | | | | | | | | |
| Sex | | | | | | | | 63 participants were female (72%) | | | | | | | | | | | | | |
| Country | | | | | | | | Malaysia | | | | | | | | | | | | | |
| Ethnicity | | | | | | | | Asian | | | | | | | | | | | | | |
| **4. Nature of intervention;** | | | | | | | |  | | | | | | | | | | | | | |
| Total number of intervention groups | | | | | | | | 2 groups | | | | | | | | | | | | | |
| Intervention specifics (sufficiency for replication, if feasible) | | | | | | | | Intervention: Certified Registered Dieticians taught basic principles of low-calorie diet (reduction of 500 kcal from estimated total calorie intake) and encouraged individuals to follow their weight reduction Worksite weight management program dietary menu which focused in reducing calorie from total fat intake. Sugars and simple carbohydrates were discouraged, and participants were taught how to ensure taking sufficient 20-30 gram of dietary fibre by: two servings of instant oatmeal together with two servings of toasted flakes of corn breakfast cereals as breakfast or dinner; one-two servings of high fibre wheat cereal crackers as snacks; including one serving of fruit in main meal; and four-five servings of leafy vegetables in a day. Participants were supervised by trained ZUMBA instructor to perform a circuit-style of high intensity interval dance, approximately 45-60 minutes with three days per week throughout the 12-week study. A patient-centred, tailored MI counselling sessions were delivered by trained RDs by exploring ambivalence and eliciting self-directed change talk. Participant were given appointment for their face-to-face cognitive consultation once a month, approximately 20-30 minutes, to identify their motive to change. Typical strategies adopted by counsellors to build motivation in those ambivalent behaviour change included agenda setting and exploration of pros and cons in negotiating a change plan.  Control: Received traditional counselling regarding diet in the form of pre-printed material by Medical Officer. These handouts were based on Ministry of Health diet and lifestyle  recommendations. They were taught about the aerobic exercise and supervised by an exercise physiologist for attending at least three sessions/week, 30-45 minute each session during the 12-week study. Facility-based exercise consisted of treadmill walking and stationary cycling; while variety of home exercises were encouraged including walking and cycling. Activity logs were reviewed weekly by investigator to monitor adherence. Participants who were not meeting target were contacted by the investigator to discuss their barrier and approaches. Specifically, this group was not interfered by any dietary (personal goal setting) or MI for behaviour support by Registered dietitians. | | | | | | | | | | | | | |
| Session content- | | | | | | | | | | | | | | | | | | | | | |
| number of sessions | | | | | | | | 3 | | | | | | | | | | | | | |
| type | | | | | | | | Face to face | | | | | | | | | | | | | |
| time | | | | | | | | 20-30 minutes each | | | | | | | | | | | | | |
| characteristics of the clinician who delivered the intervention | | | | | | | | Trained registered dieticians | | | | | | | | | | | | | |
| Reported intervention elements using TIDieR and MI checklist | | | | | | | | \| 1. Brief name \| 2. Why \| 3. What materials \| 4. What procedures \| 5. Who provided \| 6. How \| 7. Where \| 8. When and how much \| 9. Tailoring \| 10. Modifications \| 11. Planned strategies to maintain fidelity \| 12. Extent to which intervention was delivered as planned \| \| --- \| --- \| --- \| --- \| --- \| --- \| --- \| --- \| --- \| --- \| --- \| --- \| \| + \| + \| + \| + \| + \| + \| + \| + \| + \| - \| - \| - \| \| 1. Evocation \| 2. Developing a change plan \| 3. compassion \| 4. affirmation \| 5. profound acceptance \| 6. Open-ended questions \| 7. reflection \| 8. Rolling with resistance \| 9. Eliciting and strengthening change talk \| 10. Summarization \| 11. Recognizing and reinforcing change talk \| 12. Consolidating a client's commitment \| \| + \| + \| - \| - \| - \| - \| - \| - \| + \| - \| - \| - \| | | | | | | | | | | | | | |
| **5. Type of outcomes measured;** | | | | | | | |  | | | | | | | | | | | | | |
| Time points of collection and reporting | | | | | | | | 3 months | | | | | | | | | | | | | |
| Definition of outcome, unit of measurements used | | | | | | | | BMI (kg/m2), Blood Pressure (mmHg) | | | | | | | | | | | | | |
| **6. Relevant findings/results;** | | | | | | | | Intervention | | | | | | | | | | | Control | | |
| Number of participants allocated to each intervention group | | | | | | | | Patients  n=70 | | | | | | | | | | | Patients  n=70 | | |
| Sample size of each outcome, | | | | | | | | Patients=43 | | | | | | | | | | | Patients=45 | | |
| Details about missing participants | | | | | | | | non-compliance towards physical activity (16; respectively), medically unfit  (four obtained study leave and two pregnant; two transferred to district clinic, Klinik Kesihatan Kapit and one diagnosed with acute kidney failure, respectively) and high stress  level (five and seven, respectively) | | | | | | | | | | | 15, unclear  7 | | |
| Lost to follow rate | | | | | | | | 37% | | | | | | | | | | | | | |
| Changes from baseline Mean (+/- SD) for each intervention group | | | | | | | | Intervention | | | | | | | | | | | Control | | |
| Lipid profile | | | | | | | |  | | | | | | | | | | |  | | p-value |
| Total cholesterol (mmol/l) | | | | | | | | 1.34 +/- 1.45 (p-value <0.01) -22.7% | | | | | | | | | | | 0.06+/-0.69 (p-value 0.56) -1% | | |
| LDL Cholesterol (mmol/l) | | | | | | | | 1.28 +/- 1.34 (p-value <0.01) (-30.5%) | | | | | | | | | | | 3.7 +/-1.6 (p-value <0.01) [–11 %) | | |
| Triglycerides (mmol/l) | | | | | | | | 0.18+/- 0.80 (p-value <0.14) (-15%) | | | | | | | | | | | 0.66 +/- 1.22 (p-value <0.01) (47.1%) | | |
| Systolic Bp mmHg (+/-SD) | | | | | | | | 4.19 +/- 11.4 (p-value <0.02) (-3.8%) | | | | | | | | | | | 0.96 +/- 7.07 (p-value <0.37) (-0.9%) | | |
| Systolic Bp Mean difference | | | | | | | | 5.14 +/- 2.02 | | | | | | | | | | | | | 0.01 |
| Anthropometric measurement status changes from baseline | | | | | | | | | | | | | | | | | | | | | |
| Overweight/Obesity | | | | | | | |  | | | | | | | | | | |  | |  |
| Weight (SD) | | | | | | | | 4.73 +/- 3.80 (p-value <0.01) (-6.8%) | | | | | | | | | | | 0.85 +/- 2.08 (p-value <0.01) (-1.1%) | | |
| Waist circumference (SD) | | | | | | | | 7.60 +/- 5.48 (p-value <0.01) (-8.4%) | | | | | | | | | | | 0.69 +/- 1.99 (p-value <0.03) (-0.8%) | | |
| Waist circumference mean difference | | | | | | | | 6.92 +/- 0.87 | | | | | | | | | | | | | 0.01 |
| **7. Miscellaneous;** | | | | | | | |  | | | | | | | | | | | | | |
| Funding source | | | | | | | | No funding | | | | | | | | | | | | | |
| Key conclusions of authors | | | | | | | | The role of RDs in adopting MI as a strategy to facilitate client’s personal motives for behaviour change is undoubtedly important in a primary healthcare setting. | | | | | | | | | | | | | |
| Comments from the study authors | | | | | | | | The major strength of the present study is the involvement of a large number of free-living adults who participated in an already established community-based clinic program  without interacting with a team of researchers (i.e. an “effectiveness” rather than “efficacy” study). | | | | | | | | | | | | | |
|  | | | | | | | |  | | | | | | | | | | | | | |

| **Characteristics of included studies** | | | | |
| --- | --- | --- | --- | --- |
| **Ismail, 2020** | | | | |
| Methods | a multicenter, randomized controlled trial | | | |
| Participants | Patients from 12 London boroughs.  Inclusion criteria: aged 40-74 years with;  CVD 10- year risk score of ≥20.0% calculated using QRisk2 (QResearch, Nottingham, UK), which is a validated predictive tool for identifying the percentage risk of having a fatal or non- fatal cardiovascular event in the next 10 years12; fluent in conversational English; and permanent UK residence.  Exclusion criteria: medical diagnosis of CVD; having a pacemaker; diabetes, kidney disease, atrial fibrillation or stroke; chronic obstructive pulmonary disease; disabling neurological disorder; severe mental illness; registered blind; housebound or resident in nursing home; unable to move about independently; more than three falls in past year; pregnancy; advanced cancer; morbid obesity (body mass index (BMI) >50 kg/m2); participating in a weight loss programme or another participant, already randomised, in the same household.  1742 participants randomized: n=697 group intervention, n=523 individual intervention, n= 522 usual care.  Mean (SD) age: 69.75(4.11) years, females= 14.5% | | | |
| Duration of follow-up | 24-month follow-up | | | |
| ***Risk of bias*** | | |  | |
| **Bias** | **Authors’ judgement** | | **Support for judgement** | |
| - Selection bias | | |  | |
| Random sequence generation | Low risk | | The study states ‘randomisation of participants was conducted by an independent Clinical Trials Unit using computer- generated randomisation blocks.’ | |
| Allocation concealment | Unclear risk | | Insufficient information to permit judgement of low risk or high risk | |
| - Performance bias | | |  | |
| Blinding of participants and personnel | unclear risk | | The study states that it is a single blinded trial, however no details were given of how researchers went about it | |
| - Detection bias | | |  | |
| Blinding of outcome assessment | Low risk | | Data was blinded for the preliminary analysis and all outcome assessments were measured objectively.  Therefore, the overall comments for this domain, since data was blinded for analysis and objective measurements were used, we do believe that this should not introduce bias, therefore the judgment is ‘low risk’ of bias | |
| - Attrition bias | | |  | |
| Incomplete outcome data | Low risk | | Intension to treat analysis was applied | |
| - Reporting bias | | |  | |
| Selective reporting | Low risk | | Sufficient information to permit judgement of ‘low risk’ | |
| **3. Participant characteristics;** |  | | | |
| Total number | 1742 | | | |
| Setting (e.g. hospital, community) | Community | | | |
| Diagnostic criteria (e.g. hyperlipidaemia, obesity) | CVD 10- year risk score of ≥20.0% | | | |
| Age | 69.75(4.11) years | | | |
| Sex | 252 participants were female (14.5%) | | | |
| Country | UK | | | |
| Ethnicity | White Caucasian | | | |
| **4. Nature of intervention;** |  | | | |
| Total number of intervention groups | 3 groups | | | |
| Intervention specifics (sufficiency for replication, if feasible) | Intervention: The theoretical framework for enhanced motivational interviewing was based on social cognitive theory, and the theory of planned behaviour which states that to change behaviour, people need to form an intention (cognition). Intention formation is influenced by: i) expected value or positive attitude; ii) subjective norm and iii) self- efficacy. The intervention was enhanced motivational interviewing which included additional behaviour change techniques. Participants received a workbook, key learning points for every session, action planning worksheets, case studies, self- monitoring diaries and a pedometer.  Control: For UC, this consisted of referrals to locally commissioned community- based weight loss, smoking cessation and/or exercise programmes. | | | |
| Session content- | | | | |
| number of sessions | 10 | | | |
| type | Face to face | | | |
| time | 40-120 minutes each | | | |
| characteristics of the clinician who delivered the intervention | Health trainers | | | |
| Reported intervention elements using TIDieR and MI checklist | \| 1. Brief name \| 2. Why \| 3. What materials \| 4. What procedures \| 5. Who provided \| 6. How \| 7. Where \| 8. When and how much \| 9. Tailoring \| 10. Modifications \| 11. Planned strategies to maintain fidelity \| 12. Extent to which intervention was delivered as planned \| \| --- \| --- \| --- \| --- \| --- \| --- \| --- \| --- \| --- \| --- \| --- \| --- \| \| + \| + \| + \| + \| + \| + \| + \| + \| - \| - \| + \| + \| \| 1. Evocation \| 2. Developing a change plan \| 3. compassion \| 4. affirmation \| 5. profound acceptance \| 6. Open-ended questions \| 7. reflection \| 8. Rolling with resistance \| 9. Eliciting and strengthening change talk \| 10. Summarization \| 11. Recognizing and reinforcing change talk \| 12. Consolidating a client's commitment \| \| - \| - \| - \| - \| - \| - \| - \| - \| - \| - \| - \| - \| | | | |
| **5. Type of outcomes measured;** |  | | | |
| Time points of collection and reporting | 12 months, 24 months | | | |
| Definition of outcome, unit of measurements used | Weight (kg), physical activity (steps/day), lipids (mmol/mol) | | | |
| **6. Relevant findings/results;** | Individual Intervention | Group intervention | | Usual care |
| Number of participants allocated to each intervention group | n=523 | n=697 | | n=522 |
| Sample size of each outcome, | Weight n= 419  PA n= 401 | Weight n= 568  PA n=543 | | Weight n=460  PA n= 439 |
| Details about missing participants | Loss by 24 months;  Withdraw n=33  Died n=2  Non-contactable n= 27  No PA data n=43 | Loss by 24 months;  Withdraw n=69  Died n=3  Non-contactable n= 34  No PA data n=26 | | Loss by 24 months;  Withdraw n=32  Died n=4  Non-contactable n= 26  No PA data n=21 |
| Lost to follow rate | 19.7% | 18.4% | | 11.9% |
| LDL-c Mean difference for each intervention group | Individual- usual care | Group- usual care | | Individual- group |
| LDL-C | 0.05 (−0.04 to 0.14) | 0.07 (−0.01 to 0.15) | | -0.02 (-0.10 to 0.07) |
| Physical activity mean difference- group and individual versus usual care | mean difference=70.05 steps, 95% ci −288.00 to 147.90 and mean difference=7.24 steps, 95% ci −224.01 to 238.50 | | | |
| Anthropometric measurement status changes from baseline | | | | |
| Weight mean difference- group versus usual care, individual versus usual care | mean difference=−0.03 kg, 95% ci −0.49 to 0.44 and mean difference=−0.42 kg, 95% ci −0.93 to 0.09 | | | |
| **7. Miscellaneous;** |  | | | |
| Funding source | Funded project | | | |
| Key conclusions of authors | Enhanced motivational interviewing did not lead to a reduction in weight or increase in physical activity compared with usual care. | | | |
| Comments from the study authors | Enhanced motivational interviewing have little impact for reducing CVD risk in people at high CVD risk when applied to the general population. This raises the question as to whether the administration of low intensity psychological techniques in lifestyle- related interventions are of any clinical benefit. | | | |
| **Characteristics of included studies** | | | | |
| **Groeneveld, 2010** | | | | |
| Methods | Individual, randomized controlled trial | | | |
| Participants | Male workers in construction industry.  Inclusion criteria: aged 18-65 years with;  Elevated risk of CVD, Framingham risk score >moderate 10-year risk of CHD with one or more risk factor/s.  816 participants randomized: n=408 group intervention, n=408 usual care.  Mean (SD) age: 46.9 (9.1) years, males= 100% | | | |
| Duration of follow-up | 24-month follow-up | | | |
| ***Risk of bias*** | | |  | |
| **Bias** | **Authors’ judgement** | | **Support for judgement** | |
| - Selection bias | | |  | |
| Random sequence generation | Low risk | | The study states ‘randomisation of participants was conducted by a research assistant using computer- generated randomisation.’ | |
| Allocation concealment | Low risk | | Blinding of investigator responsible for data analysis but not to individual participants | |
| - Performance bias | | |  | |
| Blinding of participants and personnel | High risk | | Participants were told to which intervention they were allocated | |
| - Detection bias | | |  | |
| Blinding of outcome assessment | Low risk | | Data was blinded for the analysis and all outcome assessments were measured objectively. | |
| - Attrition bias | | |  | |
| Incomplete outcome data | Low risk | | Only individuals without missing data were included for analysis | |
| - Reporting bias | | |  | |
| Selective reporting | Low risk | | Sufficient information to permit judgement of ‘low risk’ | |
| **3. Participant characteristics;** |  | | | |
| Total number | 816 | | | |
| Setting (e.g. hospital, community) | Not reported | | | |
| Diagnostic criteria (e.g. hyperlipidaemia, obesity) | Framingham CVD 10- year risk score of ≥moderate risk | | | |
| Age | 46.9(9.1) years | | | |
| Sex | male (100%) | | | |
| Country | Dutch | | | |
| Ethnicity | White Caucasian | | | |
| **4. Nature of intervention;** |  | | | |
| Total number of intervention groups | 2 groups | | | |
| Intervention specifics (sufficiency for replication, if feasible) | Intervention: Client centred counselling style using MI techniques. MI techniques used are asking open questions, summarizing, listening, supporting, and raising ambivalence. First session discussed the CVD risk profile and current health status either on diet and/or physical activity or smoking cessation. The pros and cons of changing such a behaviour was discussed, also willingness, readiness and confidence to make such change. Short- and long-term goals were discussed.  Control: received usual care, consisting of verbal and written information about their CVD risk profile. | | | |
| Session content- | | | | |
| number of sessions | 6 | | | |
| type | 3 Face to face | | | |
| time | 45-60 minutes each | | | |
| type | 4 telephone | | | |
| time | 15-30 minutes each | | | |
| characteristics of the clinician who delivered the intervention | Occupational physician/occupational nurse | | | |
| Reported intervention elements using TIDieR and MI checklist | \| 1. Brief name \| 2. Why \| 3. What materials \| 4. What procedures \| 5. Who provided \| 6. How \| 7. Where \| 8. When and how much \| 9. Tailoring \| 10. Modifications \| 11. Planned strategies to maintain fidelity \| 12. Extent to which intervention was delivered as planned \| \| --- \| --- \| --- \| --- \| --- \| --- \| --- \| --- \| --- \| --- \| --- \| --- \| \| - \| + \| + \| + \| + \| + \| + \| + \| - \| - \| + \| - \| \| 1. Evocation \| 2. Developing a change plan \| 3. compassion \| 4. affirmation \| 5. profound acceptance \| 6. Open-ended questions \| 7. reflection \| 8. Rolling with resistance \| 9. Eliciting and strengthening change talk \| 10. Summarization \| 11. Recognizing and reinforcing change talk \| 12. Consolidating a client's commitment \| \| - \| - \| - \| - \| - \| + \| + \| - \| - \| + \| - \| + \| | | | |
| **5. Type of outcomes measured;** |  | | | |
| Time points of collection and reporting | 6 months, 12 months | | | |
| Definition of outcome, unit of measurements used | Weight (kg), BMI (kg/m^2^), systolic and diastolic bp (mmHg) | | | |
| **6. Relevant findings/results;** | Individual Intervention |  | | Usual care |
| Number of participants allocated to each intervention group | n=408 |  | | n=408 |
| Sample size of each outcome, | Weight n= 261  BMI n= 261  Systolic bp n= 259  Diastolic bp n= 259 |  | | Weight n=256  BMI n= 256  Systolic bp n=257  Diastolic n=257 |
| Details about missing participants | Loss by 12 months;  n=120 | Reasons for total N=  Lost interest n= 27  Other health problems n=14  Lack of time n=26  Retired n=2  Disappointed n=2  Measurement not planned n= 6  Unknown n= 148 | | Loss by 12 months;  n=105 |
| Lost to follow rate | 29.4% |  | | 25.7% |
| Systolic bp mean difference between groups | Mean difference= -0.3 mmHg (95% CI -2.8 to 2.2) | | | |
| Diastolic bp mean difference between groups | Mean difference= -0.4 mmHg (95% CI -1.9 to 1.1) | | | |
|  |  | | | |
| Anthropometric measurement status changes from baseline | | | | |
| Weight mean difference- intervention versus usual care | Mean difference=−1.8kgs (95% CI −2.6 to 1.1) | | | |
| BMI mean difference- intervention versus usual care | Mean difference= -0.6 (95% CI -0.8 to -0.3) | | | |
| **7. Miscellaneous;** |  | | | |
| Funding source | Funded project | | | |
| Key conclusions of authors | Individual based intervention using MI techniques can result in sustained beneficial changes in body weight. | | | |
| Comments from the study authors | It would be interesting to find out the mechanisms which led to changes in the CVD precursors. | | | |
|  |  | | | |
| **Characteristics of included studies** | | | | |
| **Hardcastle, 2007** | | | | |
| Methods | Individual, randomized controlled trial using blocks of 12 and randomly allocated in the ratio of 7:5, using computer generation | | | |
| Participants | Patients from electronic database at a local health center.  Inclusion criteria: aged 18-65 years with;  At least 1 modifiable risk factor.  552 participants randomized: n=334 group intervention, n= 218 usual care.  Mean (SD) age: 51.30(0.94) years, females= 67% | | | |
| Duration of follow-up | 6-month follow-up | | | |
| ***Risk of bias*** | | |  | |
| **Bias** | **Authors’ judgement** | | **Support for judgement** | |
| - Selection bias | | |  | |
| Random sequence generation | Low risk | | The study states ‘randomization of participants was conducted by an independent statistician using computer- generated randomization blocks of 12.’ | |
| Allocation concealment | Unclear risk | | Insufficient information to permit judgement of low risk or high risk | |
| - Performance bias | | |  | |
| Blinding of participants and personnel | Low risk | | The study states that it is a single blinded trial, where the practice nurse was blind to the treatment allocated to each patient at baseline and all subsequent assessments. Details were given of how researchers went about it | |
| - Detection bias | | |  | |
| Blinding of outcome assessment | Low risk | | Data was blinded at baseline and all subsequent assessments and all outcome assessments were measured objectively.  Therefore, the overall comments for this domain, since data was blinded for analysis and objective measurements were used, we do believe that this should not introduce bias, therefore the judgment is ‘low risk’ of bias | |
| - Attrition bias | | |  | |
| Incomplete outcome data | Low risk | | Intent to treat analysis was applied | |
| - Reporting bias | | |  | |
| Selective reporting | Unclear risk | | There is insufficient information to permit judgement, and no protocol was located for this study. | |
| **3. Participant characteristics;** |  | | | |
| Total number | 552 | | | |
| Setting (e.g. hospital, community) | Community | | | |
| Diagnostic criteria (e.g. hyperlipidaemia, obesity) | At least with 1 CHD risk factor | | | |
| Age | 51.30(0.94) years | | | |
| Sex | Female (67%) | | | |
| Country | UK | | | |
| Ethnicity | White Caucasian | | | |
| **4. Nature of intervention;** |  | | | |
| Total number of intervention groups | 2 groups | | | |
| Intervention specifics (sufficiency for replication, if feasible) | Intervention: The theoretical framework for enhanced motivational interviewing was based on principles and strategies from models of psychotherapy and behaviour change theory. The intervention was adapted motivational interviewing. The key strategies and techniques used adhere to the spirit of MI. Open-ended questions and reflective listening were used to elicit expressions of concern from patients about current health status. Different strategies were used depending on an individual’s needs and readiness to change.    Control: For UC, this consisted of a health promotion leaflet. | | | |
| Session content- | | | | |
| number of sessions | 5 | | | |
| type | Face to face | | | |
| time | No record | | | |
| characteristics of the clinician who delivered the intervention | Physical Activity Specialist (PAS) and Registered Dietitian (RD) | | | |
| Reported intervention elements using TIDieR and MI checklist | \| 1. Brief name \| 2. Why \| 3. What materials \| 4. What procedures \| 5. Who provided \| 6. How \| 7. Where \| 8. When and how much \| 9. Tailoring \| 10. Modifications \| 11. Planned strategies to maintain fidelity \| 12. Extent to which intervention was delivered as planned \| \| --- \| --- \| --- \| --- \| --- \| --- \| --- \| --- \| --- \| --- \| --- \| --- \| \| - \| + \| + \| + \| + \| + \| + \| + \| + \| + \| + \| - \| \| 1. Evocation \| 2. Developing a change plan \| 3. compassion \| 4. affirmation \| 5. profound acceptance \| 6. Open-ended questions \| 7. reflection \| 8. Rolling with resistance \| 9. Eliciting and strengthening change talk \| 10. Summarization \| 11. Recognizing and reinforcing change talk \| 12. Consolidating a client's commitment \| \| - \| + \| - \| + \| - \| + \| + \| + \| + \| - \| + \| + \| | | | |
| **5. Type of outcomes measured;** |  | | | |
| Time points of collection and reporting | 6 months | | | |
| Definition of outcome, unit of measurements used | Weight (kg), BMI (kg/m^2^), systolic Bp (mmHg), diastolic Bp (mmHg), LDL-c (mmol/l), total physical activity (met-minute/week), vigorous physical activity (met-min/week), moderate physical activity (met-min/week), walking (met-min/week), fruit and vegetable intake (portions/day) | | | |
| **6. Relevant findings/results;** | Intervention |  | | Usual care |
| Number of participants allocated to each group | n=203 |  | | n=131 |
| Sample size of each outcome, | n= 203 |  | | n= 131 |
| Details about missing participants | n/a |  | | n/a |
| Lost to follow rate | n/a |  | | n/a |
| LDL-c Mean difference for each group | Intervention |  | | Usual care |
| LDL-C | 0.09 (−0.04 to 0.37) |  | | 0.25 (-0.04 to 0.37) |
| total physical activity (met-minute/week) | 245 (-739 to 4.70) |  | | -122 (-739 to 4.70) |
| Vigorous physical activity (met-minute/week) | 149 (138 to 150) |  | | 50 (138 to 150) |
| Moderate physical activity (met-minute/week) | 89 (-358 to 121) |  | | -29 (-358 to 121) |
| Walking (met-minute/week) | 198 (-592 to -94) |  | | -145 (-592 to -94) |
| Fruit and vegetable intake (portions/day) | 1.05 (-1.36 to 0.72) |  | | 0.73 (-1.36 to 0.72) |
| BMI (kg/m^2^) | -0.21 (0.07 to 0.64) |  | | 0.15 (0.07 to 0.64) |
| Weight (kg) | -0.70 (0.02 to 1.51) |  | | 0.12 (0.02 to 1.51) |
| Systolic Bp (mmHg) | -2.90 (-o.13 to 4.62) |  | | -0.60 (-o.13 to 4.62) |
|  |  | | | |
| Funding source | Funded project | | | |
| Key conclusions of authors | Multiple sessions of adapted MI increased physical activity levels in obese females which in turn reduced weight, blood pressure and cholesterol levels. | | | |
| Comments from the study authors | An MI adapted intervention delivered by physical activity specialist and dieticians via a primary care setting can be effective to reduce CVD risk. | | | |
|  |  | | | |
| **Characteristics of included studies** | | | | |
| **Koelewijn-van Loon, 2009** | | | | |
| Methods | a multicenter, randomized controlled trial using block randomization | | | |
| Participants | Patients from 25 general practices.  Inclusion criteria: one or more CVD risk factors.  Exclusion criteria: Established CVD or familial hyperlipidaemia.  615 participants randomized: n=322 group intervention, n=293 usual care.  Mean (SD) age: 57 (7) years, females= 55% | | | |
| Duration of follow-up | 3-month follow-up | | | |
| ***Risk of bias*** | | |  | |
| **Bias** | **Authors’ judgement** | | **Support for judgement** | |
| - Selection bias | | |  | |
| Random sequence generation | Low risk | | The study states ‘cluster randomization of 25 general practices and 615 patients which was conducted by an independent statistician and using randomization blocks.’ | |
| Allocation concealment | Unclear risk | | Insufficient information to permit judgement of low risk or high risk | |
| - Performance bias | | |  | |
| Blinding of participants and personnel | Low risk | | Single blinded trial. Participants were informed about the aim of the study but not to which intervention they were allocated to. | |
| - Detection bias | | |  | |
| Blinding of outcome assessment | Unclear risk | | Insufficient information to permit judgement | |
| - Attrition bias | | |  | |
| Incomplete outcome data | Low risk | | Intension to treat analysis was applied | |
| - Reporting bias | | |  | |
| Selective reporting | Low risk | | Sufficient information to permit judgement of ‘low risk’ | |
| **3. Participant characteristics;** |  | | | |
| Total number | 615 | | | |
| Setting (e.g. hospital, community) | Community | | | |
| Diagnostic criteria (e.g. hyperlipidaemia, obesity) | CVD risk/s | | | |
| Age | 57 (7) years | | | |
| Sex | Female (55%) | | | |
| Country | Netherlands | | | |
| Ethnicity | White Caucasian | | | |
| **4. Nature of intervention;** |  | | | |
| Total number of intervention groups | 2 groups | | | |
| Intervention specifics (sufficiency for replication, if feasible) | Intervention: risk assessment, (2) risk communication, (3) distribution of a Decision support tool (DST) and (4) adapted MI. Emphasis was put on reflection about the information received in the first consultation  Control: UC, this consisted of risk assessment and ‘usual nurse led care’. | | | |
| Session content- | | | | |
| number of sessions | 2 | | | |
| type | Face to face | | | |
| time | 20 minutes each | | | |
| number of sessions | 1 | | | |
| type | telephone | | | |
| time | 10 minutes | | | |
| characteristics of the clinician who delivered the intervention | Nurses | | | |
| Reported intervention elements using TIDieR and MI checklist | \| 1. Brief name \| 2. Why \| 3. What materials \| 4. What procedures \| 5. Who provided \| 6. How \| 7. Where \| 8. When and how much \| 9. Tailoring \| 10. Modifications \| 11. Planned strategies to maintain fidelity \| 12. Extent to which intervention was delivered as planned \| \| --- \| --- \| --- \| --- \| --- \| --- \| --- \| --- \| --- \| --- \| --- \| --- \| \| + \| + \| + \| + \| + \| + \| + \| + \| - \| - \| - \| - \| \| 1. Evocation \| 2. Developing a change plan \| 3. compassion \| 4. affirmation \| 5. profound acceptance \| 6. Open-ended questions \| 7. reflection \| 8. Rolling with resistance \| 9. Eliciting and strengthening change talk \| 10. Summarization \| 11. Recognizing and reinforcing change talk \| 12. Consolidating a client's commitment \| \| - \| + \| - \| - \| + \| - \| + \| - \| - \| - \| - \| - \| | | | |
| **5. Type of outcomes measured;** |  | | | |
| Time points of collection and reporting | 3 months | | | |
| Definition of outcome, unit of measurements used | physical activity (minutes/week moderate and vigorous intensity), smoking (yes), fruit (pieces/week) | | | |
| **6. Relevant findings/results;** | Individual Intervention |  | | Usual care |
| Number of participants allocated to each intervention group | n=264 |  | | n=258 |
| Details about missing participants | Loss to follow up n=40  Personal reasons or unknown |  | | Loss to follow n=27  Personal reasons or unknown |
| Lost to follow rate | 18% |  | | 12% |
| Physical activity Mean difference for each intervention group | 455 (359) |  | | 494 (385) |
| Fruit (pieces/week | 13.4 (10.2) |  | | 14.9 (12.0) |
| Smoking | 27% | Odds ratio 1.91 | | 16% |
| **7. Miscellaneous;** |  | | | |
| Funding source | Funded project | | | |
| Key conclusions of authors | No effect of the intervention on lifestyle change. | | | |
| Comments from the study authors | The intervention seems to have an effect on risk perception which might aid with decision making. However there was no differences within the two groups in lifestyle change. | | | |
|  |  | | | |
| **Characteristics of included studies** | | | | |
| **Groeneveld 2011** | | | | |
| Methods | Individual, randomized controlled trial | | | |
| Participants | Male workers in construction industry.  Inclusion criteria: aged 18-65 years with;  Elevated risk of CVD, Framingham risk score >moderate 10-year risk of CHD with one or more risk factor/s.  816 participants randomized: n=408 group intervention, n=408 usual care.  Mean (SD) age: 46.9 (9.1) years, males= 100% | | | |
| Duration of follow-up | 12-month follow-up | | | |
| ***Risk of bias*** | | |  | |
| **Bias** | **Authors’ judgement** | | **Support for judgement** | |
| - Selection bias | | |  | |
| Random sequence generation | Low risk | | The study states ‘randomisation of participants was conducted by a research assistant using computer- generated randomisation.’ | |
| Allocation concealment | Low risk | | Blinding of investigator responsible for data analysis but not to individual participants | |
| - Performance bias | | |  | |
| Blinding of participants and personnel | High risk | | Participants were told to which intervention they were allocated | |
| - Detection bias | | |  | |
| Blinding of outcome assessment | Low risk | | Data was blinded for the analysis and all outcome assessments were measured objectively. | |
| - Attrition bias | | |  | |
| Incomplete outcome data | Low risk | | Only individuals without missing data were included for analysis | |
| - Reporting bias | | |  | |
| Selective reporting | Low risk | | Sufficient information to permit judgement of ‘low risk’ | |
| **3. Participant characteristics;** |  | | | |
| Total number | 816 | | | |
| Setting (e.g. hospital, community) | Not reported | | | |
| Diagnostic criteria (e.g. hyperlipidaemia, obesity) | Framingham CVD 10- year risk score of ≥moderate risk | | | |
| Age | 46.9(9.1) years | | | |
| Sex | male (100%) | | | |
| Country | Dutch | | | |
| Ethnicity | White Caucasian | | | |
| **4. Nature of intervention;** |  | | | |
| Total number of intervention groups | 2 groups | | | |
| Intervention specifics (sufficiency for replication, if feasible) | Intervention: Client centred counselling style using MI techniques. MI techniques used are asking open questions, summarizing, listening, supporting, and raising ambivalence. First session discussed the CVD risk profile and current health status either on diet and/or physical activity or smoking cessation. The pros and cons of changing such a behaviour was discussed, also willingness, readiness and confidence to make such change. Short- and long-term goals were discussed.  Control: received usual care, consisting of verbal and written information about their CVD risk profile. | | | |
| Session content- | | | | |
| number of sessions | 6 | | | |
| type | 3 Face to face | | | |
| time | 45-60 minutes each | | | |
| type | 4 telephone | | | |
| time | 15-30 minutes each | | | |
| characteristics of the clinician who delivered the intervention | Occupational physician/occupational nurse | | | |
| Reported intervention elements using TIDieR and MI checklist | \| 1. Brief name \| 2. Why \| 3. What materials \| 4. What procedures \| 5. Who provided \| 6. How \| 7. Where \| 8. When and how much \| 9. Tailoring \| 10. Modifications \| 11. Planned strategies to maintain fidelity \| 12. Extent to which intervention was delivered as planned \| \| --- \| --- \| --- \| --- \| --- \| --- \| --- \| --- \| --- \| --- \| --- \| --- \| \| - \| + \| + \| + \| + \| + \| + \| + \| - \| - \| + \| - \| \| 1. Evocation \| 2. Developing a change plan \| 3. compassion \| 4. affirmation \| 5. profound acceptance \| 6. Open-ended questions \| 7. reflection \| 8. Rolling with resistance \| 9. Eliciting and strengthening change talk \| 10. Summarization \| 11. Recognizing and reinforcing change talk \| 12. Consolidating a client's commitment \| \| - \| - \| - \| - \| - \| + \| + \| - \| - \| + \| - \| + \| | | | |
| Definition of outcome, unit of measurements used | Leisure time physical activity (minutes/week), fruit (pieces/week), smoking (%) | | | |
| **6. Relevant findings/results;** | Intervention |  | | Usual care |
| Number of participants allocated to each intervention group | n=408 |  | | n=408 |
| Sample size of each outcome, | Leisure time physical activity n= 207  Fruit n= 207  Smoking n=80 |  | | Leisure time physical activity n= 222  Fruit n= 221  Smoking n=82 |
| Details about missing participants | n/a |  | | n/a |
| Lost to follow rate | 19.7% |  | | 11.9% |
| Leisure time physical activity (SD) | 543.4 (462.5) |  | | 529.4 (409.2) |
| Fruit | 11.7 (8.3) |  | | 11.3 (7.8) |
| Smoking | 76.3% |  | | 80.5% |
| **7. Miscellaneous;** |  | | | |
| Funding source | Funded project | | | |
| Key conclusions of authors | The lifestyle had a significant effect in smoking and fruit intake at 6 months amongst male workers at risk increased risk. | | | |
| Comments from the study authors | Counselling using motivational interviewing is effective in reducing CVD risk in people at high CVD risk. | | | |
| Data sheet for potential meta-analysis   \| Authors \| Variable \| Number in control group \| Mean value of outcome measure in control group \| CI \| SD of outcome measure in control group \| \| Number in treatment group \| Mean value of outcome measure in treatment group \| CI \| SD of outcome measure in treatment group \| Estimated between group effect \| \| --- \| --- \| --- \| --- \| --- \| --- \| --- \| --- \| --- \| --- \| --- \| --- \| \|  \| \| \| \| \| \|  \| \| \| \| \| \| \| Kouwenhoven \| Weight (kg) \| 213 \| 0.17 \| −1.44 to 1.77 \| na \| \| 271 \| − 3.12 \| −4.26 to -1.99 \| na \| − 2.16 \| \| Aadahl \| Weight (kg) \| 68 \| 0.007 \| na \| 2.2 \| \| 81 \| –0.84 \| na \| 3.1 \| na \| \| Kong \| Weight (kg) \| 45 \| -0.85 \| na \| +-2.08 \| \| 43 \| -4.73 \| na \| +-3.80 \| na \| \| Ismail \| Weight (kg) \| 522 \| na \| na \| na \| \| 523 \| na \| na \| na \| -0.42 \| \| Groeneveld \| Weight (kg) \| 256 \| 0.3 \| na \| na \| \| 261 \| -0.9 \| na \| na \| -1.2 \| \| Hardcastle \| Weight (kg) \| 131 \| 0.12 \| na \| 0.29 \| \| 203 \| -0.70 \| na \| 0.25 \| na \| \|  \| \| \| \| \| \|  \| \| \| \| \| \| \| Kouwenhoven \| BMI (kg/m^2^) \| 213 \| -0.12 \| 0.67 \| na \| \| 271 \| -1.0 \| -0.39 \| na \| − 0.81 \| \| Groeneveld \| BMI (kg/m^2^) \| 256 \| 28.5 \| na \| +-3.9 \| \| 261 \| 28.5 \| na \| 3.7 \| -0.6 \| \| Hardcastle \| BMI (kg/m^2^) \| 131 \| 0.15 \| 0.07 to 0.64 \| na \| \| 203 \| -0.21 \| 0.07 to 0.64 \| na \| na \| \|  \| \| \| \| \| \|  \| \| \| \| \| \| \| Aadahl \| Waist circumference (cm) \| 68 \| -0.24 \| na \| +-2.7 \| \| 81 \| -1.18 \| na \| +-4.0 \| na \| \| Kong \| Waist circumference (cm) \| 45 \| -0.69 \| na \| +-1.99 \| \| 43 \| -7.60 \| na \| +-5.48 \| na \| \|  \| \| \| \| \| \|  \| \| \| \| \| \| \| Aadahl \| LDL-c (mmol/l) \| 68 \| -0.06 \| na \| +-0.5 \| \| 81 \| -0.21 \| na \| 0.6 \| na \| \| Kong \| LDL-c (mmol/l) \| 45 \| -0.47 \| na \| +-1.16 \| \| 43 \| -1.28 \| na \| +-1.34 \| na \| \| Ismail \| LDL-c (mmol/l) \| 522 \| 2.94 \| na \| 0.90 \| \| 523 \| 3.02 \| na \| 0.88 \| 0.05 \| \| Hardcastle \| LDL-c (mmol/l) \| 131 \| 0.25 \| na \| 0.08 \| \| 203 \| 0.09 \| na \| 0.07 \| na \| \|  \| \| \| \| \| \|  \| \| \| \| \| \| \| Boutin \| Systolic blood pressure (mmHg) \| 87 \| na \| na \| na \| \| 90 \| na \| na \| na \| na \| \| Kong \| Systolic blood pressure (mmHg) \| 45 \| -2.44 \| na \| +-10.29 \| \| 43 \| -4.19 \| na \| +-11.40 \| na \| \| Groenevald \| Systolic blood pressure (mmHg) \| 256 \|  \| na \| +-16.2 \| \| 261 \| 138.1 \| na \| +-16.2 \| -0.3 \| \| Hardcastle \| Systolic blood pressure (mmHg) \| 131 \| -0.60 \| -0.13 to 4.62 \| na \| \| 203 \| -2.90 \| -0.13 to 4.62 \| na \| na \| \|  \| \| \| \| \| \| \| \| \| \| \| \| \| Aadhal \| Stepping time \| 68 \| na \| na \| na \| \| 81 \| na \| na \| na \| 0.15 \| \| Ismail \| Steps/day \| 522 \| na \| na \| na \| \| 523 \| na \| na \| na \| 7.24 \| \| Koelewijn-van Loon, 2009 \| Physical activity (minutes/wk) \| 258 \| 494 \| na \| +-385 \| \| 264 \| 455 \| na \| +/-359 \| na \| \| Groeneveld 2011 \| Physical activity (minutes/wk) \| 222 \| 529.4 \| na \| +-409.2 \| \| 207 \| 543.4 \| na \| +/-462.5 \| na \| \| Lin \| Physical activity (Met-min/wk) \| 39 \| na \| na \| na \| \| 38 \| na \| na \| na \| 846 \| \| Hardcastle \| Physical activity (Met-min/wk) \| 131 \| -122 \| -739 to 4.70 \| na \| \| 203 \| 245 \| -739 to 4.70 \| na \| na \| \| Groeneveld 2011 \| Physical activity (Met-min/wk) \| 223 \| 2591.1 \| na \| +-1899 \| \| 206 \| 2678.6 \| na \| +/-1838.4 \| 132.2 \| \|  \| \| \| Percentage \|  \| \| \| \| Percentage \|  \| \| \| \| Kouwenhoven \| Lack of physical activity (%) \| 213 \| -50.3 \| na \| na \| \| 271 \| -53.6 \| na \| na \| −5.6 \| \| Boveda \| Increase in physical activity (%) \| 98 \| na \| na \| na \| \| 98 \| na \| na \| na \| 23.3 \| \| Lakervald \| Meeting physical activity recommendations (%) \| 253 \| 51.9 \| na \| na \| \| 249 \| 51.6 \| na \| na \| na \| \| Koelewijn-van Loon, 2009 \| Meeting physical activity recommendations (%) \| 258 \| 71 \| 0.52 to 1.09 \| na \| \| 264 \| 64 \| 0.52 to 1.09 \| na \| na \| \|  \|  \|  \|  \|  \|  \| \|  \|  \|  \|  \|  \| \| Kouwenhaven \| Smoking cessation (%) \| 213 \| 0 \| na \| na \| \| 271 \| -3.2 \| na \| na \| -8.6 \| \| Lakervald \| Smoking cessation (%) \| 253 \| -18.3 \| na \| na \| \| 249 \| -17 \| na \| na \| na \| \| Koelewijn-van Loon \| Smoking cessation (%) \| 258 \| -3 \| na \| na \| \| 264 \| -7 \| na \| na \| na \| \| Groeneveld 2011 \| Smoking cessation (%) \| 82 \| -19.5 \| na \| na \| \| 80 \| -23.7 \| na \| na \| na \| \|  \|  \|  \|  \|  \|  \| \|  \|  \|  \|  \|  \| \| Lakerval \| Meeting fruit intake recommendations (%) \| 253 \| 22 \| na \| na \| \| 249 \| 18 \| na \| na \| na \| \| Koelewijn-van Loon \| Fruit (pieces/week) \| 258 \| 14.9 (pieces/week) \| na \| +-12 \| \| 264 \| 13.4 (pieces/week) \| na \| +/-10.2 \| na \| \| Groeneveld 2011 \| Fruit (pieces/week) \| 221 \| 11.3 (pieces/week) \| na \| +-7.8 \| \| 207 \| 11.7 (pieces/week) \| na \| +/-8.3 \| 0.9 \| \| Lakervald \| Meeting vegetable intake recommendations (%) \| 253 \| 18 \| na \| na \| \| 249 \| 19.7 \| na \| na \| na \| \|  \|  \|  \|  \|  \|  \| \|  \|  \|  \|  \|  \| \|  \| \| \| Mean value of outcome measure in control group \|  \| \| \| \| Mean value of outcome measure in intervention group \|  \| \| \| \| Kong \| Energy(kcal/day) \| 45 \| -108.2 \| na \| +-351.1 \| \| 43 \| -553 \| na \| +-339.2 \| na \| \| Kong \| Protein (g/day) \| 45 \| -5.7 \| na \| +-16.8 \| \| 43 \| -21.07 \| na \| +-14.1 \| na \| \| Kong \| Fat (g/day) \| 45 \| -18.9 \| na \| +-21.1 \| \| 43 \| -45.3 \| na \| +-16.3 \| na \| \| Kong \| Carbohydrates (g/day) \| 45 \| 19.2 \| na \| +-61.87 \| \| 43 \| -15.6 \| na \| +-47.2 \| na \| \| Kong \| Dietary ﬁber(g/day) \| 45 \| 2.7 \| na \| +-4.4 \| \| 43 \| 7.8 \| na \| +-4.3 \| na \| \|  \| \| \| \| \| \| \| \| \| \| \| \| | | | | |

**Table S5. Intervention content checklist.**

| **Reported intervention characteristics using TIDieR** | Item No | Checklist item | **Element reported in the study** | |
| --- | --- | --- | --- | --- |
|  |  |  | YES | NO |
| Brief name | 1 | Includes a name that describes the intervention |  |  |
| Why | 2 | Description of the rationale, theory or goal of the elements essential to the intervention |  |  |
| What materials | 3 | Description of materials used, including those provided to participants, or used in intervention delivery, or in training of intervention providers. Provide information of where the materials can be accessed. |  |  |
| What procedures | 4 | Description of procedures, activities, and/or processes used. |  |  |
| Who provided | 5 | Description of the expertise, background, and any specific training given of the intervention provider |  |  |
| How | 6 | Description of the modes of delivery (e.g. face-to-face, internet, telephone) and whether it was delivered individually or in a group |  |  |
| Where | 7 | Description of the location where the intervention occurred, including any necessary infrastructure or relevant features |  |  |
| When and how much | 8 | Description of the number of times the intervention was delivered and over what period of time including the number of sessions, their schedule, and their duration, intensity and dose |  |  |
| Tailoring | 9 | If the intervention was planned to be personalised, titrated and adapted, description of the what, why, when and how is included |  |  |
| Modifications | 10 | If the intervention was modified during the course of the study, description of the what, why, when and how is included |  |  |
| Planned strategies to maintain fidelity | 11 | If intervention adherence or fidelity was assessed, description of how and by whom, and the strategies used to maintain or improve fidelity |  |  |
| Extent to which intervention was delivered as planned | 12 | If intervention adherence or fidelity was assessed, description of the extent to which the intervention was delivered as planned |  |  |

**Table S6. Intervention content checklist.**

| **Motivational interviewing elements** | Item No | Checklist item | **Element reported in the study** | |
| --- | --- | --- | --- | --- |
|  |  |  | YES | NO |
| **The principles of MI** | | | | |
| Profound acceptance | 1 | Description of acceptance; e.g. clients accepted the way they are without judgements, the clinician being empathetic, supporting autonomy and not telling clients what to do |  |  |
| Compassion | 2 | Description of compassion; e.g. actively promoting the other’s welfare and giving priority to their needs. |  |  |
| Evocation | 3 | Description of evocation; e.g. the clinician attempts to evoke and strengthen the motivations to change that already exist in the client |  |  |
| **OARS** | | | | |
| Open-ended questions | 4 | Description of open-ended questions; e.g. the clinician asks questions which require the client to answer in more than few words |  |  |
| Affirmation | 5 | Description of affirmation; e.g. acknowledging the client’s inherent strengths and efforts (affirmation) |  |  |
| Reflection | 6 | Description of reflection; e.g. rephrasing the client’s words and thoughts to establish clarity |  |  |
| Summarization | 7 | Description of summarization; e.g. compiling together the main points, with a purpose to emphasize, refocus or change direction |  |  |
| **Recognizing and reinforcing change talk** | | | | |
|  | 8 | Description; e.g. listen for, elicit, reinforce, and point out change talk of the related behaviour to clients. |  |  |
| **Eliciting and strengthening change talk** | | | | |
|  | 9 | Description; e.g. use of evocative questions, asks for a description when the client has made a change statement, asking the client how they view the future, gives the client individualized feedback from an assessment and have the client interpret the meaning of the assessment, making use of readiness rulers and following it up with questioning client why did they rate themselves where they did |  |  |
| **Rolling with resistance** | | | | |
|  | 10 | Description; e.g. reflecting on what the client says, emphasizing autonomy |  |  |
| **Developing a change plan** | | | | |
|  | 11 | Description; e.g. negotiating with the client, answering questions, provide advice and information |  |  |
| **Consolidating a client’s commitment** | | | | |
|  | 12 | Description; e.g. listening for client’s commitment language and strengthening such commitment |  |  |
