# Supplementary material for: Motivational interviewing to support modifiable risk factor change in individuals at increased risk of cardiovascular disease: A systematic review and meta-analysis
Source: PLoS One. 2020 Nov 11;15(11):e0241193. doi: 10.1371/journal.pone.0241193 (PMC7657493; doi:10.1371/journal.pone.0241193)
Supplement: Attachment — Submitted filename: Response to reviewers.docx [file pone.0241193.s002.docx]

Response to reviewers.

Re: Motivational interviewing to support modifiable risk factor change in individuals at increased risk of cardiovascular disease: a systematic review and meta-analysis. (Reference number PONE-D-19-35907)

Dear Editor,

I would like to thank the reviewers for their helpful comments. I have taken their suggestions on board and did all the possible changes as discussed below. Please find enclosed the edited manuscript in word format. I believe that now the manuscript is suitable for publication.

Editorial comments

When submitting your revision, we need you to address these additional requirements:

1. Please ensure that your manuscript meets PLOS ONE's style requirements, including those for file naming.

Reply: Thank you. We have reviewed PLOS ONE’s style requirements and have edited our manuscript as necessary.

2. We note that your systematic review search is restricted to articles published until August 2018. Please ensure that your search is up to date and the systematic review/meta-analysis includes any studies published since then.

**Reply:** The search was updated till March 2020.

3. We note that you have indicated that data from this study are available upon request. PLOS only allows data to be available upon request if there are legal or ethical restrictions on sharing data publicly.

**Reply:** All completed data collection sheets are now available in S1 Appendix.

Reviewer’s feedback (reviewer 1)

4. *The abstract is structured using appropriate sub-headings and is concisely written. The initial background section states MI has not been well established and explored (line 56), which is a little misleading because there is a wealth of research investigating the use of MI with various health settings and the paper later refers to a recent systematic review on a similar topic. It might be worth adding some additional context to this statement e.g. what areas are poorly established and have not been explored?*

**Reply:** Thank you for pointing this out. Now I have clarified that this statement is specifically referring to primary prevention. This review, gives focus on individuals without pre-existent cardiovascular disease.

5. *The introduction sets the scene for the review and outlines the role of lifestyle therapies in the prevention of CVD. The introduction also introduces MI and outlines some of the core features of the approach. Understandably this is a fairly concise overview of MI, but I wonder whether it would be worth adding something about how these features are used when working with ambivalence and supporting behaviour change?*

**Reply:** Thank you for asking this question. I have elaborated on the application of open questions, affirmation, reflective listening, summarizing, informing and advising. This now shows how these features are applied to address ambivalence and support behavior change.

6. *The introduction continues by highlighting a gap in the literature and stating how this review compliments other literature. The paper then provides a separate rationale section, which is a little unusual. I wonder whether this rationale content might be better embedded within the introduction section?*

**Reply:** Thank you for the comment. The rational content is now embedded within the introductory section.

7. *The review question is appropriate, but could be a little concise (e.g. Our review sought Line 141). There is also a little blurring between effectiveness, strategies, and mechanisms. I wonder whether it might be clearer to present the aims as three bullet points: 1) effectiveness 2) characteristics 3) mechanisms?*

**Reply:** Thank you for this point. Amendments were done as follows:

‘Our review sought to determine the effectiveness of MI intervention in supporting primary prevention through changing modifiable cardiovascular risk factors. Additionally, the review provides an account of the characteristics of MI interventions reported in trials that supported risk factor modification.

The primary and secondary review questions are as follows:

1) Is MI effective in supporting adults at increased risk of cardiovascular disease to make healthy lifestyle changes to reduce cardiovascular risk?
2) What are the characteristics of MI interventions that support risk factor modification?
8. *The methods section begins by stating the review aligns with PRISMA, which is good. Although, I could see the attached table in the supporting information section to check completeness. A search strategy section is provided, but this could be more transparent and possibly more accurate. The search strategy is rather difficult to read because of the way the information is presented. The use of brackets to group the databases accessed via EBSCO is useful, but this is not used for databases accessed via ProQuest. I could be wrong, but I also think there is some confusion between databases (e.g. MEDLINE) and database providers (e.g. EBSCO and ProQuest) because there is not a consistent approach to how these are presented. It might be useful to list the databases and put the provider in brackets (e.g. MEDLINE (ProQuest)).*

*The use of key words/subject headings could also be more explicit and it is unclear whether Boolean operators and/or delimiters were used. I understand an example of the search strategy is provided in the supplementary information, but I was unable to access this. I would also suggest changing the term* *“concepts” (line 164) to “search terms” or similar.*

**Reply:** The search was retaken. Table showing details (results for each systematic step, use of Boolean operators, truncations and wildcard symbols, application of filters) is now available for each database. The term “concepts” was used when referring to the main term (Coronary heart disease, Motivational interviewing, Adult, Prevention, Risk modification, and Clinical trial). For each concept then there are a number of search terms. For example for Coronary heart disease; "coronary disease*" OR "cerebrovascular disorder*" OR "cardiovascular disease*" OR "cardiovascular disorder*" OR "cerebrovascular disease*" OR "heart disease*" OR "myocardial infarction" OR "heart disease*" OR "coronary*disease" OR "ischemic*disease" OR "athero*" OR "myocardial"….these all fall under this concept. Search strategy is now included in Table S3 of S1 appendix

9. *The study selection section refers to RCTs “a high body of evidence”, which is rather unusual language (line 185) and might benefit from re-wording.*

**Reply:** We thank the reviewer for pointing this out. The sentence now reads as follows:

Studies for inclusion were those published between February 2013 and March 2020, but limited to randomised controlled trials as reliable sources of evidence.

10. *The data extraction section makes reference to critical appraisal (lines 205-208), but this is rather confusing and might be better re-worded and placed within the risk of bias section further down the paper. (Page 6)*

**Reply:** We thank the reviewer for the suggestion. This was now amended and moved under the risk of bias section.

11. *The paper includes a study outcomes sub-heading, which might be unnecessary given the other text provided. This section also does not mention effectiveness, which is one of the main aims of the review.*

**Reply:** We thank the reviewer for the suggestion. We feel that this section now shows the specifics and it was amended as follows 235-250:

Effectiveness of the intervention using MI was determined by change in modifiable cardiovascular risk factors (smoking status, dietary eating patterns, physical activity levels, lipid profile levels, blood pressure levels, weight, waist circumference, body mass index).

Data on the characteristics of the MI interventions designed to support CVD risk factor modification were identified;

1) Reported intervention characteristics:

- Nature of the intervention
- Type, frequency, duration, interval
- Characteristics of the deliverer (professional discipline, training and experience)
- Setting

2) Reported MI elements:

- Motivational interviewing content of the intervention

12. *The results section then provides a summary of the study characteristics. It would be good to explain the differences in the RCTs (line 265), so readers learn a little more about the studies instead of having to refer to the table. Table two shows the study characteristics, but could be enhanced by providing details of the actual participants (e.g. gender, mean age) instead of reporting the inclusion/exclusion criteria used in the original studies.*

**Reply:** I thank the reviewer for this suggestion. Table 2 was made more explicit. Exclusion criteria was removed and replaced by details such as gender and mean age. More details were included about the RCT design. Line 292-295.

13. *The effectiveness of MI in supporting modifiable risk factor change is reported comprehensively. A summary of MI component is also provided, but I am a little unsure how the 12 items were selected and wonder whether a more comprehensive reporting method might be better (e.g. TIDieR DOI: 10.1136/bmj.g1687). It might also be worth considering whether the studies being reviewed used any competence measures (e.g. MITI DOI: 10.1016/j.jsat.2004.11.001)..*

**Reply:** We thank the reviewer for this comment. Now the TIDieR checklist is referred to under the subheading ‘Reported intervention elements’ and table 4. line 451-454.

We do not feel that the TIDieR should replace our previous work, but added to it. Table 5. also reflects to items covered by the TIDieR checklist. These then were categorized according to the study methodological qualities. This will support clinicians/researchers to select intervention components which look promising and likely to succeed. The motivational interviewing content reported was assessed against a checklist that was developed by the authors and drew upon literature from Miller and Rollnick (see Table S6 in S1 appendix). We believe that this highlights the lack of intervention elements reported and the importance of using an MI competence measure.

14. *The discussion and limitation section are clear and make relevant points. The conclusion is brief and accurate. Check wording (line 606) and consider amending “possess”.*

**Reply:** I thank the reviewer for their comment. This was now changed to ‘adapted’.

Reviewer2:

15. *Pg 3 line 63-64: It is not clear why only manuscripts from 2013 to August 2018 were explored. What about earlier studies or studies done since after August 2018?*

**Reply:** I thank the reviewer for the comment. Two similar systematic reviews were taken previously. These included searches from January 1999 to December 2009, and from 1980 to February 2013. The difference is that our review has specifically focused on individuals without pre-existent CVD. Now we have updated the search up to March2020.

16. *Introduction: The rationale for this review was not effectively established. Given that a previous review suggested primary research (page 5lines 116 – 118) why implement a review instead of a primary research.*

**Reply:** We thank the reviewer for the comment. The previous review included RCTs up to February 2013, and did not specifically focus on individuals without pre-existent disease. It was decided that firstly a systematic literature review should be conducted. Our review identified 8 RCTs, which were critically appraised using the Cochrane risk of bias tool. Suggestions for a primary study have been put forward throughout this review. A protocol for a primary study is being developed and a primary research study will be tested in the future.

17. *Rationale Page 6 line 125 – 127 (The impact of MI… has remained suboptimal) – provide citation*

**Reply:** I thank the reviewer for the comment. Now citations are provided.

18. *Search strategy- page 7 It is not clear if the search strategy is adequate. The readers will benefit from search strategy/ies implemented in individual database/s and journal/s. EbSCO host a number of databases so a generic strategy cannot apply to all the databases in EBSCO. I wonder what informed the decision to do a separate search in BMJ and Plos Journals and not the numerous psychology and allied health journals. It is also strange that BMJ and Plos journals will return lots of articles whereas search in databases returned just a few numbers. It was not clear where the conference proceedings were searched (169)*

**Reply:** I thank the reviewer for pointing this out. Search was retaken. Searches were done individually. We have included a Table (see Table S3 in S1 appendix) showing details of the filters applied and the search results. The search strategy section in the manuscript was amended accordingly.

19. *Study Selection –pg 8Line 183 – what does the authors mean by international studies? Line 185… Not clear what high body of evidence means. Lines 190 – 191… Reference lists of identified studies were manually search to identify further eligible publication - at what point was this done? Was this done only for the 7 included papers? How was disagreement between reviewers regarding studies eligibility dealt with? What was done where it was not declared in a study whether or not people with established CVD were included?*

**Reply:** We thank the reviewer for the comments. What we meant by ‘international studies’ is, all studies across the globe. However, since this might not have been interpreted clearly, we decided to remove the term ‘international’.

By having a high body of evidence, we were referring to RCT design. This was amended and now reads line 204-206;

‘but limited to randomised controlled trials as these are initially considered as the gold standard of evidence (11, 12)’.

Manual searching of reference lists was done for the 8 identified RCTs.

Any disagreements between the two researchers, were solved through discussion and if necessary, the third researcher was consulted. All studies declared clear inclusion criteria. Only one study was excluded after consulting with the third researcher. This study included participants who underwent coronary angiography. Such a procedure will only be done in symptomatic individuals (positive Exercise tolerance stress test, stable angina, unstable angina, ECG changes). For the latter reason, it was decided to exclude the study and list under ‘secondary prevention’.

20. Data extraction – pg 9 Lines 205- 207 … Could this be stated as one of the limitations? Only 3/7 satisfied the items on the risk of bias tables. Again, consideration should be given to the relevance of each of the items to the overall risk of bias outcomes. E.g. Is it possible to blind a personnel delivery MI to group allocation?

**Reply:** We thank the reviewer for the comments. The following sentence; ‘In our review, critical appraisal for methodological quality was not done to ascertain eligibility status of the studies to be included, but to identify intervention effectiveness of those studies who have used a high-quality methodology’, was now moved under the subheading ‘Risk of bias assessment and quality of evidence’.

We do understand that it is not always possible to blind personal. Such interventions are considered as ‘complex interventions’, hence a double blinded procedure is not always possible. However, we feel that the risk of bias outcome, does reflect the features of the design and conduct of the included studies.

21. Risk of bias assessment and quality of evidence – pg 10 It is great that assessment of risk of bias was done at the outcome level but were there considerations to the relevance of each domain to individual outcomes? Could GRADE rating be objectively executed when there not formal meta-analysis in majority of the outcomes? How was inconsistency, imprecision, indirectness and publication bias assessed?

**Reply:** We thank the reviewer for the comments.

We have reconsidered how to go about the individual outcomes. Now the GRADE’s approach was used for meta-analysis (LDL-c, weight) only. We started with a high default rating, since the included studies are RCTs.

For these 2 outcomes (LDL-c, weight) a decision was made whether to downgrade or upgrade the certainty of the evidence by one or two levels for each factor, leading to a final rating.

The level of certainty was downgraded by one level for serious concerns about risk of bias or two levels for very serious concerns. The I^2^ statistic, was used for the meta-analysis, and indicated high statistical heterogeneity for the two outcomes (LDL-c, weight). The results for these outcomes across the studies were consistent and directing towards effectiveness. Therefore, the high statistical heterogeneity could be explained by the variations between the interventions used. This could be seen as a clear reason for heterogeneity. Therefore, it was decided not to downgrade, hence reason why ‘inconsistency’ for ‘Improved LDL-c’ and ‘Decreased weight’ were marked as ‘not serious’. For ‘decreased weight’, as the confidence interval excluded the possibility of no effect, we are confident that an effect is present, so we decided that imprecision was not serious. On the other hand, for ‘Improved LDL-c’, the result was considered imprecise as the confidence interval includes both a meaningful result in one direction and crosses the line of no effect. Also, the confidence interval resulted wide, therefore cannot exclude any meaningful effect in either direction. Hence, the ‘Improved LDL-c’ outcome was downgraded by one level for serious concerns about imprecision. We decided not to downgrade for indirectness, however we made it clear that the results only refer to the population reflected in the systematic review (individuals at an increased risk of CVD).

Publication bias was addressed with the Cochrane RoB (risk of bias) tool. This was considered under the risk of bias/study limitations factor for GRADE.

22. Data synthesis – pg 10

There are three aspects of heterogeneity – conceptual/clinical, statistical, and methodological. Some aspect of methodological heterogeneity may have been dealt with by including only RCT. However, MI in this review were part of broader interventions in different contexts. Therefore, transparent reporting and justification of how data are grouped for synthesis is essential to completely deal with the methodological and conceptual heterogeneity. Synthesis of results- Apart from the implemented meta-analysis, there were no real attempt at synthesis of results but a mere corroboration of the results from primary studies, making this section bulky and cumbersome to understand. In addition, implementing alternative metric synthesis means alternative questions were answered. Although, this was mentioned in passing in the data synthesis section, the authors did not reflect this in reporting result synthesis. Indeed, meta-analysis asks the question of what is the size of the average effect. On the other hand, methods like summarising effect estimates only answer the question of the range and distribution of the effect. Combining p-values will show if there is evidence that there is an effect in at least in one study. Lastly, vote counting based on direction (trends) of an effect teases out if there is evidence of an effect? Therefore, the manuscript will benefit from a real synthesis and reporting that recognises these differences.

**Reply:** We thank the reviewer for the comments.

For each outcome (numerical), we planned to extract:

Number in control group

Mean value of outcome measure in control group

SD of outcome measure in control group

Number in treatment group

Mean value of outcome measure in treatment group

SD of outcome measure in treatment group

A number of outcomes common to 2 or more studies (weight, LDL cholesterol) were observed. Meta analyses were conducted with respect to both of these outcomes. Although the pre-specified outcomes are all measurable outcomes, it was not possible to quantitively synthesize most of the outcomes from the different studies. This is due to either existent and substantive differences in how the outcomes were measured across the studies; substantive differences in study parameters outwit reasonable limits of heterogeneity, or unavailable statistical information. Where possible, certain parameters, which were not provided, were calculated from others that were given: e.g. SD was calculated from mean, confidence interval and number of cases. However, for most of the variables we did not have the means of generating the statistics needed for a particular study for both study groups being compared Therefore we could not include the study in an MA.

All outcomes that could not be quantitatively synthesized were interpreted narratively.

We conducted meta analyses using the more conservative random effects models (rather than fixed effects models), reflecting the clinical and methodological diversity between the studies considered, which is identified and acknowledged elsewhere in our paper. The statistical heterogeneity established in the meta analyses is likely to reflect this observed clinical and methodological diversity and suggests that the utilisation of random effect models was appropriate

23. Summary of outcome findings

Similar to synthesis of results, the summary of outcomes findings lumps everything together. For instance, it is not clear what was being compared against MI for each of the outcomes – MI vs other behavioural change techniques, or MI vs usual care. In addition, the interpretation of the quality of evidence did not reflect that each of the synthesis were not from a meta-analysis (as per the diver synthesis methods).

**Reply:** We thank the reviewer for the comments. We have amended this section. Now references are made available for each of the outcomes. The quality of evidence now reflects the outcomes from the meta-analysis.

24. Reviewer 1: *Overall, this is a really interesting topic and thorough systematic review. It will be of interest to a wide number of people and help inform practice/research. It does need some minor revision before publication and could be written more concisely in places.*
25. *Reviewer 2: Many thanks for inviting to review this manuscript. Overall, the topic is interesting but there are numerous weaknesses, lack of clarity, poor synthesis of results and general inconsistencies throughout the manuscript that made it difficult to review it to the end. I have picked at sections and will go ahead to provide some comments. I am happy to re-review the whole manuscript when the authors have attended to the preliminary comments. The manuscript will immensely benefit from professional editing service prior to resubmission.*

**Reply:** We thank the reviewers for finding the topic and the results interesting to a wide number of health professionals to support better practice and research in preventive cardiology.

I would be glad to answer any further questions or comments that you may have.

I look forward to hearing from you,

Thank you for your time,

Justin Lee Mifsud
