# Supplementary material for: Motivational interviewing to support modifiable risk factor change in individuals at increased risk of cardiovascular disease: A systematic review and meta-analysis
Source: PLoS One. 2020 Nov 11;15(11):e0241193. doi: 10.1371/journal.pone.0241193 (PMC7657493; doi:10.1371/journal.pone.0241193)
Supplement: Attachment — Submitted filename: Response to reviewers.docx [file pone.0241193.s003.docx]

**Revision 2.**

Author response

Justin L. Mifsud – Department of Nursing, University of Malta

Email address: justin-lee.mifsud@um.edu.mt

ORCID iD: <https://orcid.org/0000-0001-5380-9418>

Re: Motivational interviewing to support modifiable risk factor change in individuals at increased risk of cardiovascular disease: a systematic review and meta-analysis. (Reference number PONE-D-19-35907)

Dear Editor,

I would like to thank you and the reviewers for their helpful comments. I have taken their suggestions on board and did all the possible changes as discussed below. Please find enclosed the edited manuscript in word format. We believe that now the manuscript fully meets PLOS ONE’s publication criteria.

Comments to the Author

1. If the authors have adequately addressed your comments raised in a previous round of review and you feel that this manuscript is now acceptable for publication, you may indicate that here to bypass the “Comments to the Author” section, enter your conflict of interest statement in the “Confidential to Editor” section, and submit your "Accept" recommendation.

Reviewer #1: All comments have been addressed

Reply: Thank you. We have now addressed all the points highlighted by reviewer 2 and reviewer 3.

2. Is the manuscript technically sound, and do the data support the conclusions?

The manuscript must describe a technically sound piece of scientific research with data that supports the conclusions. Experiments must have been conducted rigorously, with appropriate controls, replication, and sample sizes. The conclusions must be drawn appropriately based on the data presented.

Reviewer #1: Yes

Reviewer #2: Yes

Reviewer #3: Yes

Reply: Thank you

3. Has the statistical analysis been performed appropriately and rigorously?

Reviewer #1: I Don't Know

Reviewer #2: Yes

Reviewer #3: Yes

Reply: Thank you

4. Have the authors made all data underlying the findings in their manuscript fully available?

The PLOS Data policy requires authors to make all data underlying the findings described in their manuscript fully available without restriction, with rare exception (please refer to the Data Availability Statement in the manuscript PDF file). The data should be provided as part of the manuscript or its supporting information, or deposited to a public repository. For example, in addition to summary statistics, the data points behind means, medians and variance measures should be available. If there are restrictions on publicly sharing data—e.g. participant privacy or use of data from a third party—those must be specified.

Reviewer #1: Yes

Reviewer #2: Yes

Reviewer #3: Yes

Reply: Thank you

5. Is the manuscript presented in an intelligible fashion and written in standard English?

PLOS ONE does not copyedit accepted manuscripts, so the language in submitted articles must be clear, correct, and unambiguous. Any typographical or grammatical errors should be corrected at revision, so please note any specific errors here.

Reviewer #1: Yes

Reviewer #2: Yes

Reviewer #3: Yes

Reply: Thank you

*Reviewer #2: Authors have done well to address almost all my queries. However, I continue to argue that given the two previous reviews did not specifically focus on people without pre-existing CVD, findings from them should not represent results specific to people without pre-existing condition. I therefore suggest extending the search to older literature which focused on people without pre-existing CVD.*

Reply: Thank you, we have now extended the search and included older studies which fit the inclusion and exclusion criteria of our review.

*Reviewer #3: interesting paper.*

Reply: Thank you

1. *Abstract*

*Definition of Cochrane framework should be added*

Reply: Thank you, we have now included the principles of the Cochrane framework.

2. *Why from 8 studies for only 3 was analysis performed?*

Reply: Thank you for your comment. In view of variations between studies in measuring the variables, meta-analysis was not always possible.

3. *Authors say that some features may be more likely like ...: how were these variables defined?*

Reply: Thank you for your comment. We have deemed these particular intervention characteristics as potential intervention items, after carrying out a careful evaluation of risk of bias and intervention effectives of each study. Studies which had minimal risk and showed significant intervention benefits, their intervention characteristics were identified and selected as more likely to be effective than other characteristics. The selected characteristics will then be used in primary research.

4. *Introduction is too long and should be shortened*

Thank you for your comment, we have now deleted some of the word count in the introduction section.

5. *Methods: did authors performed sub analysis according to grading of paper*

Reply: By “sub analysis” we assume the reviewers are referring to subgroup analyses as part of the meta analyses. These were not conducted as there were no appropriate strata in the included studies over which results could be presented. We chose to assess statistical heterogeneity with random effects meta analyses.

6. *Methods: authors spoke about conservative effect for random. This should be better specified*

Reply: Thank you for your comment. More detail is now included in the manuscript. We conducted meta analyses using the more conservative random effects models (rather than fixed effects models), reflecting the clinical and methodological diversity between the studies considered, which is identified and acknowledged elsewhere in our paper. The statistical heterogeneity established in the meta analyses is likely to reflect this observed clinical and methodological diversity and suggests that the utilisation of random effect models was appropriate.

7. *Results: plots should be modified and made more consistent*

Reply: Thank you for your comment. We have now modified the plots to include data from the new studies. The plots have been, and remain consistently presented, and they and the accompanying summary text follow a standard format for the presentation of meta analyses results.

Editorial comments

1. *Abstract: In the Conclusion section that is a result - please remove that.*

Reply: Thank you for pointing this out. We have edited the conclusion section and moved part of it under the result section.

2. *Main body of the manuscript: line 113 you refer to one systematic review and meta-analysis - please be clear about your meaning here.*

Reply: Thank you for pointing this out. We were referring to the same study which is a systematic review with meta-analysis. Now this has been clarified in the manuscript.

3. *One review is about primary and secondary prevention, the other is not. Line 130 you refer to updating previous reviews - this is not accurate - the focus of your current review is narrower, please clarify.*

**Reply:** Thank you for pointing this out. Now we have made it explicitly clear that our review specifically focuses on primary prevention. Our review, gives focus on individuals without pre-existent cardiovascular disease. For this reason, we agreed to update the search and included older studies which were eligible as per inclusion/exclusion criteria.

4. *Line 161 after ‘electronic journals’ is everything in brackets an electronic journal or did you look at electronic journals within these resources? Please clarify.*

**Reply:** Thank you for pointing this out. Now we have clarified this sentence in the manuscript. This now reads- The search strategy was formulated and applied to identify published primary research literature from databases (CINAHL Complete, APA PsycINFO, Academic Search Ultimate, Cochrane Central Register of Controlled Trials, MEDLINE, PubMed,) and electronic journals within health-related resources (E-Journals, Wiley Online Library, PLOS, DynaMed Plus).

5. *Line 164 I agree with the reviewers, the use of the word ‘concept’ is not useful here. It would be clearer to explain the PICOs elements (to use the term you use in Table 1) and then describe your search in terms of the PICOs elements.*

**Reply:** Thank you for pointing this out. Now we have replaced the term concept by the term ‘search terms’ which links to the PICO elements.

6. *Line 180 are these ‘preventive interventions’ primary prevention interventions? Please clarify.*

**Reply:** Thank you for your comment. We are referring to primary prevention interventions, therefore we now have included the term ‘primary’.

7. *Line 185 remove the word ‘but’ and replace it with ‘and’*.
8. *Line 191 is it possible to conduct searches using Endnote? If not, remove the words ‘retrieved and’.*
9. *Line 192 insert the word ‘potentially’ between the 'further’ and ‘eligible’.…..*

**Reply:** Thank you for the suggestions. We now have amended the manuscript accordingly.

10. *Table 1 insert ‘primary’ in your description of the Inclusion criteria for the Intervention element.*

*line 212 I am aware that the reviewers suggested that you use the TIDieR checklist. The review would be more robust, I think, if you did used TIDieR to report the data extracted. You can add supplementary items, if you felt this was necessary but I haven't identified any items in sections 1) or 2) that would be supplementary. All would fit with TIDieR, item four, in particular. Also, I note you have used TIDieR in the Results section therefore it would make sense to also use it here.*

**Reply:** We have now referred to the TIDieR under the heading ‘methods’. S1 appendix now shows items checked for each individual study. This is then presented in Table 4. ‘Summary of reported intervention elements’. We used percentage scoring for each item and calculated the overall mean, as we believe that this will make it more interesting for PLOS readers.

Now we have used TIDieR to report intervention items as shown under the subheading ‘Secondary outcomes- Reported intervention elements.

Then we moved on to report The MI intervention element. The item checklist to check for MI elements is review specific and draws upon literature from Miller WR, Rollnick S, (Motivational interviewing: Helping people change: Guilford press; 201 2). The results of the checklist used is now available for each study as part of the S1 appendix. Table S5 is the template of the MI checklist. We believe that this checklist highlights the lack of intervention elements reported and the importance of using an MI competence measure.

11. *Lines 226 and 228 - swap the order of these two sentences. Line 223 do you mean ‘data extraction’ rather than ‘data abstraction’? Move the sentence to the data extraction section above and state that it was review specific or ‘adapted from...’*

*line 250 the heterogeneity paragraph in this location should be about methods only. The results element of this paragraph belongs in the Results section.*

**Reply:** Thank you for the suggestions. We have now amended the manuscript accordingly.

12. *Results: Line 256 you report 1114 hits which after duplicates were removed reduced to 860. 792 records were then excluded – leaving a total of 68 - NOT 69 as stated. This mistake is replicated in the PRISMA flow diagram. Please check the numbers and amend both the text and the flow diagram accordingly.*

**Reply:** Thank you, we have now re-taken the search to extend to older studies. We have changed numerical records accordingly.

13. *Table 2-Aadahl (2014) record complete the comment in the ‘control’ section. Remove ‘both’ from after ‘gender’ in the Boutin-Foster, (2016) record. Insert 100% after ‘females’ in the Lin (2016) record. Insert key to acronyms used in the table*

**Reply:** Thank you for the suggestions. We have now amended the table accordingly.

14. *Line 303 the meaning here is unclear – either there was or wasn’t selective reporting.*

**Reply:** Thank you for pointing this out. We have clarified this statement and it now reads: There was no selective reporting in 6 of the 12 studies

15. *Line 306 surely there was reporting bias in what was reported (Tabel 3 indicates that this is the issue) and, if that is the case, it is not clear why you have singled out one study for comment.*

**Reply:** Thank you for your comment. We have commented on 1 particular study, as this study had a protocol available, however it was noted that not all pre-specified outcomes were reported in a pre-specified way. Therefore, this study can be indicative of selective reporting. The remaining 6 studies did not provide sufficient detail about the reporting of study outcomes as no protocol was available. Therefore, judgement with respect to reporting bias could not be carried out.

16. *Line 312 replace ‘into’ with ‘to; ‘by’ is not the right word to use in this sentence. Line 314 – provide an example. Line 322 remove ‘existent and’ Line 324 ‘outwith’ not ‘outwit’ Line 330 here and for all outcomes headings remove the information* *in brackets from the heading. Line 368 – consider reporting this is such a way as to demonstrate that it is a positive finding.*

**Reply:** Thank you for the suggestions. We have now amended the manuscript accordingly.

17. *Line 414 why was a meta-analysis not doe for BP? After a bit of digging, I find it was because the results were reported in different was – it may be worth explaining that here – but also please note, the references are numbered incorrectly. Please check all reference numbering.*

**Reply:** Thank you for the suggestions. We have now amended the manuscript accordingly. Meta-analysis was not always possible due to variations between studies in the measurements used to calculate same variable/s. Also, we were not able to use the Groeneveld study in the revised meta analysis of weight. This was because although Groeneveld measured weight in both groups at baseline and at 12 months, no stats were provided for the change in weight. Of course, mean weight change can easily be calculated from baseline and follow-up data, but the SD of weight change cannot. We were able to add the other studies, and create new plots accordingly.

18. *Line 459 - what papers are being referred to in this section?*

**Reply:** Thank you for pointing this out. We are referring to the studies by Kouwenhaven 2018, Aadahl, 2014 and Kong, 2017. Reference is being made in a forest plot (fig. 3), showing that the synthesized data from the three studies, overall result favour the intervention in reducing weight.

19. *Lines 479 – 481 How do you justify your interpretation of this finding? Is 73% ‘adequate’?*

**Reply:** Thank you for pointing this out. This has now changed since we have included older studies. Now the percentage is 68%. This now reads:

The average of total percentage reporting to at least one of the 12 items across all 12 studies amounted to 68%, highlighting that the overall intervention descriptions were adequately reported (Table 4) and may, support replicability of the intervention.

20. *Table 4 The meaning of the second row from the bottom is unclear I.e. Mean overall (%) reporting rate to at least one element*

**Reply:** Thank you for pointing this out. We have now amended this in the manuscript. This now reads: Mean overall (%) reporting

Line 505 I think there is typo - ‘elements’ is used twice, and the meaning is unclear.

**Reply:** Thank you, this is now corrected.

21. *Indicators paragraph*

*This paragraph needs work the English could be improved and the paragraph would perhaps benefit from an explanatory opening sentence. For example, my understanding is that frequency of use of MI elements is mapped against intervention effectiveness. Is that right? This should be described in the methods section and not be making a first appearance here.*

**Reply:** Thank you for your comment. Our intension was to identify and select intervention program elements from those studies which showed a significant beneficial between group difference effect. Only elements from high quality studies were selected. The use of these intervention elements was then put forward to be used in primary research. A protocol for a primary study has been drafted and is proposed to be tested.

22. *Table 5 Presenting this aspect of your findings in this way is a good idea but I would be tempted to reduce this to include only the high-quality study columns - as these will be the results most likely to influence future practice, surely?*

**Reply:** Thank you for your suggestion. We have now included only the elements from high quality studies.

23. *The limitations section needs to include the issue of using English language papers only as this is not considered good practice.*

**Reply:** Thank you for your comment. This is now included in the limitations section.

**Reply:** We thank the reviewers for finding the topic and the results interesting to a wide number of health professionals to support better practice and research in preventive cardiology.

I would be glad to answer any further questions or comments that you may have.

I look forward to hearing from you,

Thank you for your time,

Justin Lee Mifsud
